# Direct Amplification, Sequencing and Profiling of *Chlamydia trachomatis* Strains in Single and Mixed Infection Clinical Samples

**DOI:** 10.1371/journal.pone.0099290

**Published:** 2014-06-27

**Authors:** Sandeep J. Joseph, Ben Li, Tanvi Ghonasgi, Chad P. Haase, Zhaohui S. Qin, Deborah Dean, Timothy D. Read

**Affiliations:** 1 Department of Medicine, Division of Infectious Diseases, Emory University School of Medicine, Atlanta, Georgia, United States of America; 2 Department of Biostatistics and Bioinformatics, Rollins School of Public Health, Emory University, Atlanta, Georgia, United States of America; 3 Center for Immunobiology and Vaccine Development, Children's Hospital Oakland Research Institute, Oakland, California, United States of America; 4 Department of Human Genetics, Emory University School of Medicine, Atlanta, Georgia, United States of America; 5 Department of Medicine, University of California San Francisco, San Francisco, California, United States of America; 6 Joint Graduate Program in Bioengineering, University of California San Francisco, San Francisco, California, United States of America; 7 University of California Berkeley, Berkeley, California, United States of America; Auburn University, United States of America

## Abstract

Sequencing bacterial genomes from DNA isolated directly from clinical samples offers the promise of rapid and precise acquisition of informative genetic information. In the case of *Chlamydia trachomatis*, direct sequencing is particularly desirable because it obviates the requirement for culture in mammalian cells, saving time, cost and the possibility of missing low abundance strains. In this proof of concept study, we developed methodology that would allow genome-scale direct sequencing, using a multiplexed microdroplet PCR enrichment technology to amplify a 100 kb region of the *C. trachomatis* genome with 500 1.1–1.3 kb overlapping amplicons (5-fold amplicon redundancy). We integrated comparative genomic data into a pipeline to preferentially select conserved sites for amplicon design. The 100 kb target region could be amplified from clinical samples, including remnants from diagnostics tests, originating from the cervix, urethra and urine, For rapid analysis of these data, we developed a framework for whole-genome based genotyping called *binstrain*. We used *binstrain* to estimate the proportion of SNPs originating from 14 *C. trachomatis* reference serotype genomes in each sample. Direct DNA sequencing methods such as the one described here may have an important role in understanding the biology of *C. trachomatis* mixed infections and the natural genetic variation of the species within clinically relevant ecological niches.

## Introduction

*Chlamydia trachomatis* is an obligate intracellular bacterial parasite that causes both ocular and sexually transmitted infections (STIs) in humans worldwide. Ocular infections lead to the disease trachoma, which is the leading global cause of preventable blindness [1]. Despite its importance as a human pathogen, significant technical barriers to research have hindered progress, notably the lack of a genetic system to probe gene function, although advances have been made in the past few years [2]–[4]. Because of the difficulty of working with the organism, whole genome sequencing of multiple strains over the past 15 years has had a profound impact on our understanding of *C. trachomatis* biology[5]–[11].

*C. trachomatis* requires expensive, time- and labor-intensive cell culture for *in vitro* growth. This has been a major technical roadblock in the production of pure genomic DNA, and makes large scale comparative genome studies using cheap sequencing technologies much more difficult to achieve than bacterial pathogens that can be cultured in cell-free systems. The dependence on cell culture necessarily involves generations of plaque purification (e.g., to ensure segregation of clonal populations of *C. trachomatis* for genome sequencing) with intermittent population bottlenecks. The final product of multiple rounds of culture may be genetically different from the strain causing the clinical infection [11]. The time and expense of plaque purification (or even non-plaque cultures) has restricted the number of *C. trachomatis* strains that have been collected over the years. Moreover, not all strains of *C. trachomatis* can successfully be cultured from clinical samples, even when the bacterium is detected by a commercial diagnostic or in-house assay. Recently there has been progress in developing culture free sequencing for *C. trachomatis*. Antibodies attached to magnetic beads were used to pull down *C. trachomatis* cells from the milieu in clinical samples prior to whole genome amplification and sequencing [12], [13]. While the preliminary results were promising there were significant amounts of carryover non-chlamydial DNA in the output sequence. Furthermore, the antibody technique could not be used on remnant swab samples from most commercial NAATs (nucleic acid amplification tests) since the latter use a lysis buffer that destroys the chlamydial membrane, which is a target for the antibody. Thus, many *C. trachomatis* positive clinical samples would not be available for genomic analysis using this approach.

Here, we describe an alternative system for extracting DNA directly from clinical samples (both original and remnant media used for commercial NAATs), enriching the *C. trachomatis* DNA by specific PCR, and performing genome sequencing. Our approach makes use of the PCR-based micro-droplet platform introduced by RainDance Technologies (Lexington, MA) [14]. In this proof of concept study, we generated targeted genome sequences of *C. trachomatis* directly from DNA purified from isolates and from clinical urine and clinical urethral and cervical swab samples. We utilized single nucleotide polymorphism (SNP) information from existing genome projects along with nucleotide coverage data obtained at each aligned SNP position to reliably identify the diversity of single or mixed *C. trachomatis* strains in both the simulated and real clinical sample data.

## Materials and Methods

### *C. trachomatis* samples, DNA extraction, *ompA* genotyping and MLST

We used three sets of genomic DNA (gDNA) samples in this study. The first set (Set 1) included nine samples each of 10 ng gDNA extracted from purified, cultured Elementary Bodies (EBs) that we had previously genome sequenced [7], [11] (Table 1). Set 2 consisted of 14 samples of 20 ng of gDNA each extracted from clinical urogenital samples (urine, cervix and urethra). The cervical and urethral samples in this set had been collected and placed into either 1 mL of SPG or M4 buffer (in SPG or M4 buffer, MicroTest, Inc.); 100 µL of the buffer were tested by the Roche Amplicor NAAT (Roche Diagnostics) as per the package insert and the 14 samples were found to be positive for *C. trachomatis*. Additionally, 500 µL of the remaining M4 buffer was used for culture, and 400 µL of the remaining buffer were used to extract gDNA using the commercial Roche High Pure Kit (Roche) as described previously [15]. Set 3 consisted of 10 Amplicor NAAT positive clinical urethral and cervical swab samples in either SPG or M4 buffer as above, each of 20 ng gDNA extracted using the commercial QIAamp DNA Micro Kit (Qiagen) after elution of cellular material from the remnant clinical swab sample into 100 µl of ddH_2_O. Elution was required to obtain the cells containing the chlamydial EBs for subsequent DNA purification. DNA samples were quantified and visualized for purity on an Agilent 2100 Bioanalyzer. *ompA* genotyping and MLST (Multi-locus sequence typing) were performed on the extracted gDNA using previously described techniques [15]–[17].

**Table 1:** Details of the *C. trachomatis* samples along with the preliminary analysis results that were successfully amplified in this study.

Sample ID	*ompA*/MLST	Sample source	Total reads	No. reads mapped	No. reads failed to align	No. reads aligned to target 100 kb region	No. of reads that aligned outside of the targeted 100 kb region	Mean Coverage	Major genotypes (*binstrain* β>0.05)
**Set 1 (n = 9)**		DNA from culture							
1.1 (E/5s)	E/Ja	cervix	8573090	7077055 (82.55%)	1496035 (17.45%)	7024734 (81.94%)	52321 (0.73%)	3567.4	F, Da, E
1.2 (Ja/47nl)	Ja/E	cervix	5476167	5120882 (93.51%)	355285 (6.49%)	5078095 (92.73%)	42787 (0.83%)	2574.9	F, Ja
1.3 (C/TW-3/OT)	C/C	eye	8319753	7670255 (92.19%)	649498 (7.81%)	7613236 (91.5%)	57019 (0.74%)	3897.6	C
1.4 (H/UW-4/CX)	H/H	cervix	7332997	6852494 (93.45%)	480503 (6.55%)	6802203 (92.76%)	50291 (0.73%)	3448.02	H
1.5 (L2C)	L2	rectum	1846853	1657112 (89.73%)	189741 (10.27%)	1644277 (89.03%)	12835 (0.77%)	850.55	L2
1.6 (L2C -Not Sheared)	L2	rectum	12049537	10887857 (90.36%)	1161680 (9.64%)	10798045 (89.61%)	89812 (0.82%)	5509.23	L2
1.7 (Clinical D)	D/E	cervix	5941072	5551015 (93.43%)	390057 (6.5%)	5504198 (92.65%)	46817 (0.84%)	2802	E, Da, F
1.8 (Clinical D - Not Sheared)	D/E	cervix	8514764	7936110 (93.20%)	578654 (6.8%)	7872318 (92.5%)	63792 (0.80%)	4008.7	E, Da, F
1.9 D/UW-3/CX	D	cervix	3239426	2789213 (86.10%)	450213 (13.90%)	2769186 (85.48%)	20027 (0.71%)	2790.19	D
**Set 2 (n = 14)**		DNA from remnant NAAT							
2.1 (Clinical F)	F/F	cervix	10	na	na	na	na	na	na
2.2 (Clinical Ia)	Ia/Ia	cervix	10	na	na	na	na	na	na
2.3 (Clnical K)	K/K	cervix	9982911	5867295 (58.77%)	4115616 (41.23%)	5832182 (58.42%)	35113 (0.59%)	5595.4105	Ia, J
2.4 (Clinical E)	E/Da	cervix	5824277	1604620 (27.55%)	4219657 (72.45%)	1595635 (27.40%)	8985 (0.55%)	1736.91	E, A, L2, G
2.5 (Clinical D)	D/D	cervix	10	na	na	na	na	na	na
2.6 (Clinical F)	F/F	urethra	10	na	na	na	na	na	na
2.7 (Clinical E)	E/E	cervix	1476979	552932 (37.44%)	924047 (62.56%)	549844 (37.23%)	3088 (0.55%)	590.89	E
2.8 (Clinical E)	E/E	cervix	10	na	na	na	na	na	na
2.9 (Clinical E)	E/E	urethra	10	na	na	na	na	na	na
2.10 (Clinical E)	E/Da	cervix	9129274	4370021 (47.87%)	4759253 (52.13%)	4349449 (47.64%)	20572 (0.47%)	4059.7	F, Da, E
2.11 (Clincal I)	I/I	cervix	10	na	na	na	na	na	na
2.12 (Clinical J)	J/J	urine	10	na	na	na	na	na	na
2.13 (Clinical F)	F/F	urine	10	na	na	na	na	na	na
2.14 (Clinical F)	F/Ja	urine	11270048	8925527 (79.20%)	2344521 (20.80%)	8866904 (78.68%)	58623 (0.65%)	6553.87	F, Da
Set 3 (n = 10)		DNA from remnant NAAT							
3.4 (Clinical Ja + F (1∶1))	Ja/F	cervix	8533744	8071019 (94.58%)	462725 (5.42%)	7994126 (93.68%)	76893 (0.95%)	6297.05	Ja, F, Da
3.5 (Clinical Ja + F (1∶5))	Ja/F	cervix	6983664	6657861 (95.33%)	325803 (4.67%)	6600229 (94.51%)	57632 (0.86%)	5732.61	F, Ja, Da
3.7 (Clinical Ja + F (1∶50))	Ja/F	cervix	3434780	3269121 (95.18%)	165659 (4.82%)	3243409 (94.43%)	25712 (0.78%)	3309.9	F, Da, Ja
3.6 (Clinical F)	F/F	cervix	10	na	na	na	na	na	na
3.15 (Clinical Ia)	Ia/Ia	urethra	8358088	7878533 (94.26%)	479555 (5.74%)	7814947 (93.50%)	63586 (0.80%)	6358.7	Ia, J
3.16 (Clinical F)	F/F	urethra	6947384	6362388 (91.58%)	584996 (8.42%)	6308372 (90.80%)	54016 (0.85%)	5697.6	F, Da
3.17 (Clinical Ia)	Ia/Ia	urethra	14952564	13884185 (92.85%)	1068379 (7.15%)	13780473 (92.16%)	103712 (0.75%)	7511.24	Ia, J
3.18 (Clinical E)	E/E	cervix	12662104	11384620 (89.91%)	1277484 (10.09%)	11287132 (89.14%)	97488 (0.85%)	7012.27	F, E, Da
3.19 (Clinical D)	D/F	cervix	9285480	6619031 (71.28%)	2666449 (28.72%)	6579106 (70.85%)	39925 (0.60%)	5532.75	F, Da
3.20 (Clinical Ia)	Ia/Ia	urethra	10	na	na	na	na	na	na

1 Droplet PCR amplification failed.

### Microdroplet Amplification

A 500 primer pair microdroplet library was synthesized by RainDance Inc. The primer library and a mix that included the template DNA and all the components of the PCR reaction excluding the primers were loaded separately on a RainDance RDT 1000 instrument for merging. In preliminary experiments, we found that as little as 0.5 ng purified gDNA template would produce up to 250 ng post-amplification DNA. For the work described in this manuscript, we used 10 or 20 ng of input DNA. The merged droplets were then amplified using an Applied Biosystems 9700 thermocycler with the following conditions: 94°C for 2 minutes, 55 cycles at 94°C for 15 seconds, 54°C for 15 seconds and 68°C for 10 minutes, with a hold at 4°C. After amplification, the PCR droplet emulsion was broken to release the DNA amplicons. The amplicon was then purified using a Promega clean up kit and quantified using the 2100 Bioanalyzer. The bioanalyzer trace was also inspected to verify that the expected amplicon peak was in the 1000–1100 nt range.

### Sequencing amplified DNA

One to two micrograms of micro-droplet post-amplification DNA was used to make sequencing libraries for the Illumina Hi-Seq 2000 instrument, using Beckman reagents. Prior to library construction, DNA was sheared to 300 bp+/−30 bp using a Covaris E300L acoustic focusing instrument. Subsequent steps were performed on the Beckman robot with reagents specially designed for Hi-Seq libraries. Each Illumina library was tagged with one of 12 oligonucleotide barcodes. The libraries were quantified using KAPA (Woburn, MA) qPCR quantitation kits on a Roche Lightcycler and quality checked using an Agilent Bioanalyzer. Each lane was loaded with up to 10 multiplexed libraries and run for 100 nt reversible terminator sequencing cycles in a single direction.

### Simulated sequence data

Simulated 100 bp Illumina read sequence data was generated using ART software [14], [18]. ART simulated sequencing reads by mimicking the output of the Illumina sequencing process with empirical error models summarized from large sets of recalibrated sequencing data. FASTQ files were generated based on 13 *C. trachomatis* reference strains (Table S1 in File S1) with published and non-published genome sequences [6], [7], [9], [11] at coverage similar to that obtained in real experiments (1000× to 7000×). To simulate mixed cultures, we merged two or more synthetic single strain FASTQ files. We also generated FASTQ files based on previously identified 6 *C. trachomatis* recombinant strains identified at MLST loci (Figure S1 in File S1).

### Phylogenetic reconstruction

The whole genome core alignment and the core alignment of the targeted 100 kb region were extracted from a MAUVE alignment of 14 *C. trachomatis* whole genome (Table S1 in File S1) in order to estimate the phylogenies. Both phylogenies were estimated using the GTR substitution model by the neighbor joining (NJ) method implemented in NEIGHBOR in the PHYLIP package [19] as well as using the maximum likelihood approach using the PhyML program. The support of the data for each of the internal node of the phylogeny was estimated using 100 bootstraps.

### *C. trachomatis* ancestral sequence regeneration

An ancestral core genome sequence of *C. trachomatis* was generated using the baseml program in the PAML package [20] and used as an estimate for a baseline comparison to modern *C. trachomatis* for SNP calling (see below). For generating the ancestral sequence of *C. trachomatis* (CT_ASR), baseml was implemented using a whole genome alignment of 8 *C. trachomatis* genomes (Table S1 in File S1) representing all 4 clades of *C. trachomatis* phylogeny [7], and the corresponding whole genome phylogenetic tree. The genomes (with NCBI accession numbers) used were: D/UW3/CX (genbank:AE001273), L2/434/BU(genbank:AM884176), A/HAR-13(genbank:CP000051), B/TZ1A828/OT (genbank:FM872308), E/11023 (genbank:CP001890), F/70 (genbank:ABYF01000001), G/9301 (genbank:CP001930), L2b/UCH-1 (genbank:AM884177) (Table S1 in File S1). We implemented GTR nucleotide substitution model, with 5 gamma rate categories, assuming that the model was homogeneous across all the sites.

### Sequence data analysis

Sequence reads generated from the RainDance experiment and simulated data for each sample were mapped against the *C. trachomatis* reference genome (D/UW-3/CX) and CT_ASR genome using Burrows-Wheeler transform (BWA) short-read aligner [21] by specifying the maximum number of gap extensions (e) to be 10. The resultant short-read alignment files for each samples were converted to mpileup format using the mpileup option in SamTools software [22] along with the –B option that disables probabilistic realignment for the computation of base alignment quality (BAQ). Average read depth (Coverage) mapped to the reference genome for the 100 kb region for each of the amplified samples is shown in Table 1. To calculate the Major Allele Percentage (MAP) at each site, we filtered the mpileup table for all positions with at least 100-fold coverage, and for each position divided the called base with the highest number of reads by the sum of all the called bases.

## Results

### Ascertainment of *C. trachomatis* genotypes in simulated pure and mixed cultures

**Figure 1:**
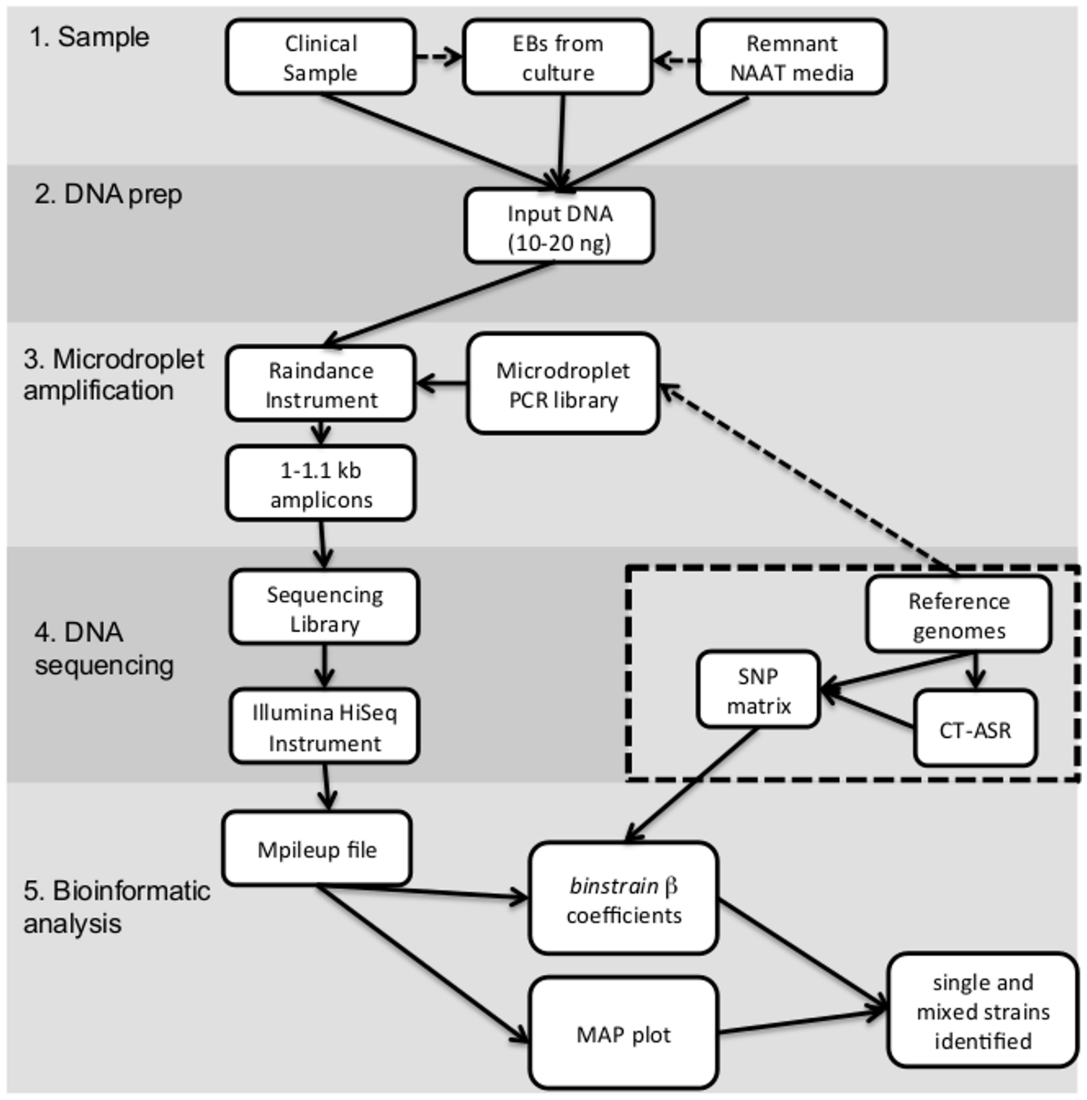
Flow chart representing the workflow of the entire procedure of target amplification, sequencing and genotype profiling performed in this study. The dashed box represents the core analysis starting with downloaded reference genomes necessary for primer design and *binstrain* analysis. CT-ASR is the *C. trachomatis* ancestral reconstruction sequence.

The steps in the analysis described in this study are summarized in Figure 1. Before development of direct sequencing methods, we addressed two primary challenges in downstream analysis of the resulting data: 1) determination of whether there was more than one *C. trachomatis* genotype present in the sample, and 2) estimation of the genotypes of the component strain(s) in the respective sample. We created simulated FASTQ files with various proportions of coverage of the target 100 kb genome region (see next section) for 13 *C. trachomatis* reference strains and 6 additional clinical strains. We also created single, bi-mixture and tri-mixture strains by merging FASTQ files (Table S2 in File S1).

**Figure 2:**
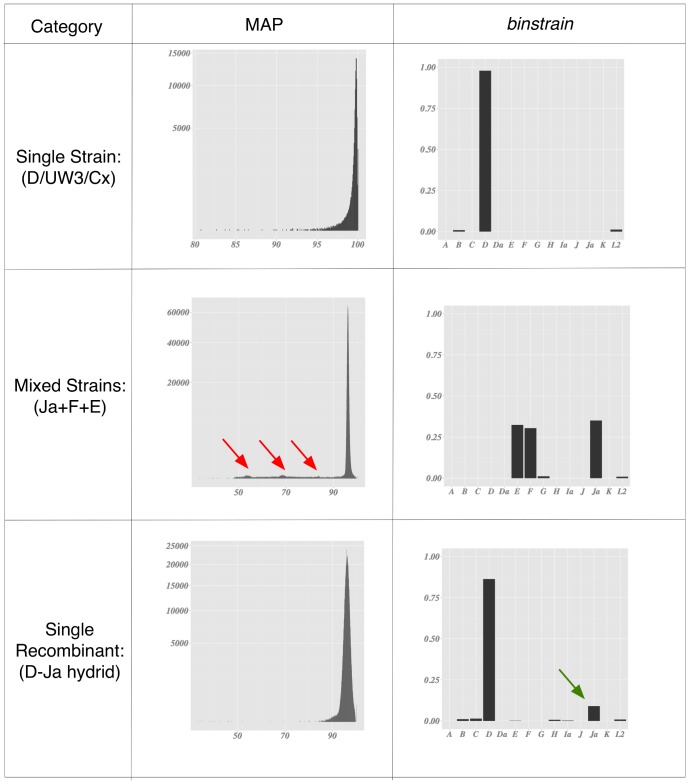
Summary of *binstrain* analysis of selected simulated data. Each panel illustrates representative data for (top to bottom) a single strain identical (or very similar) to a previously sequenced genome, a mixture of strains, and a single novel recombinant strain. From left to right are the histogram of Major Allele Percentage (MAP) across the sequenced region and a barplot of *binstrain* **β** values for the reference genome set. In the MAP plots, minor peaks representing the subpopulation of mixed alleles are shown by red arrows. The minor **β**-value associated with the introduction recombinant strain is shown with a green arrow.

In order to detect mixed strain cultures, we plotted a statistic termed here as ‘Major Allele Percentage’ (MAP), defined as the percentage of the most common nucleotide at each position of the sequence read mpileup table (with an arbitrary minimum cutoff of samples with at least 100× coverage redundancy). In the simulated data (Figure 2; Figures S4, S6, S8, S10, S12 and S14 in File S1) the overwhelming majority of positions had a MAP in the 96%+ range (the noisiness in the data was due to the error model used in the simulation). In the mixed strain simulated data, concentrations of sites above the background noise with MAP less than 95% could be visualized graphically, representing loci where there are a mixture of alleles (Figures S4 and S6 in File S1). Clusters of MAP loci <96% are missing in single strain simulations.

While MAP could identify mixed-strain samples, it did not provide feedback on genetic constitution. In order to provide a rapid analysis of the template genotype from target capture experiments, we developed a program (*binstrain*) that used a binomial mixture model to predict the most likely genetic background(s) of the *C. trachomatis* sample. Traditional genotyping methods such as Multiple Locus Sequence Typing (MLST) were not developed to deal with potential mixtures of strains in unknown proportions [23]. The *binstrain* algorithm also made use of information from across the entire target region, rather than being limited to a small number of genes. In developing *binstrain*, we assumed a binomial probability distribution, *p_i_* of observing an alternative allele (SNP) in the targeted region at position *i*:
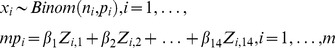
where *m* indicates the number of SNP positions in the targeted region or entire genome depending on the experiment, *n_i_* denotes the number of total read coverage at position *i*, and *x_i_* denotes the number of alternative alleles at position *i. Z_ij_* is an indicator function specifying whether *j* ^th^ strain has an alternative allele at *i^th^* position. The estimation of *β_j_* indicates the proportion of strain-specific SNPs present in a clinical or purified sample. At the strain–specific SNP positions, there would be only a few *β_j_*s that affects *p_i_*_._ Other *β_j_*s have no impact on *p_i_* because their corresponding *Z_ij_* are 0's, which makes it a sparse design matrix. We utilized this sparsity of the design matrix in order to perform a well-established step-by-step procedure to estimate all the *β_j_*s. In the first step, we estimated as many *β_j_*s as possible by utilizing the sparsity of the design matrix at all the strain-specific SNP positions (rows on the matrix) and, if the estimate for *β_j_* is less than 0.05, we treated those *β_j_*s as 0 in the following estimate so that the unknowns can be reduced. After excluding all those *β_j_*s, we used quadratic programming, an optimization method, to handle the remaining *β_j_*s in the second step. This optimization method ensured that the *β_j_*s are non-negative. The algorithm was implemented as an R package, named “*binstrain*”, publicly available at https://github.com/benliemory/BinStrain.

**Figure 3:**
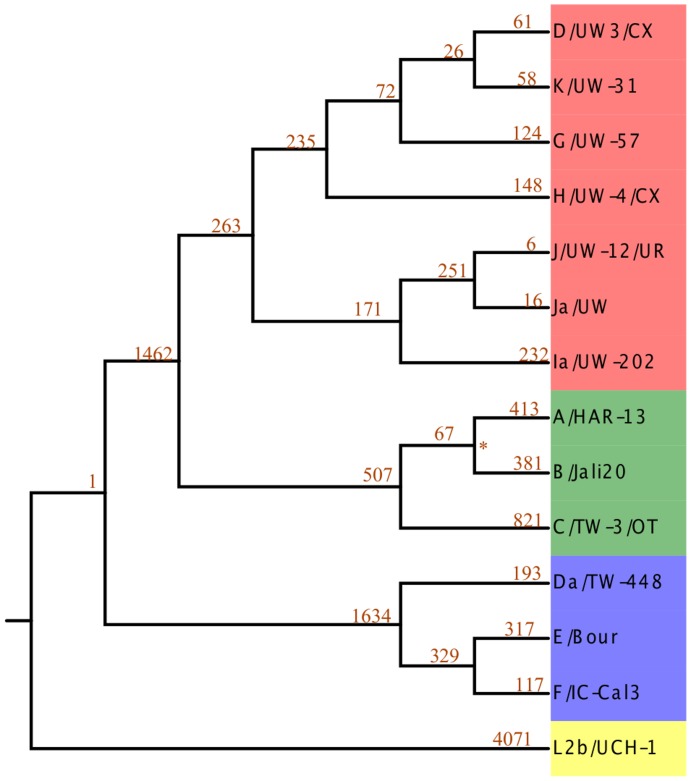
Whole-genome phylogeny of the reference *C. trachomatis* strains used in this study. The tree was constructed using a neighbor-joining algorithm based on whole-genome alignment. All the branches were supported by 100% confidence in 100 bootstrap sampling except for the A/HAR-13/B/Jali20 branch. All nodes with bootstrap support <100% are designated with an asterisk. Each leaf and internal branch has the number of SNPs unique to this branch compared to the CT-ASR, reconstructed ancestral sequence. The leaves are colored by membership of major *C. trachomatis* Clades [7]: yellow, blue, green and red for Clades 1–4, respectively.

**Figure 4:**
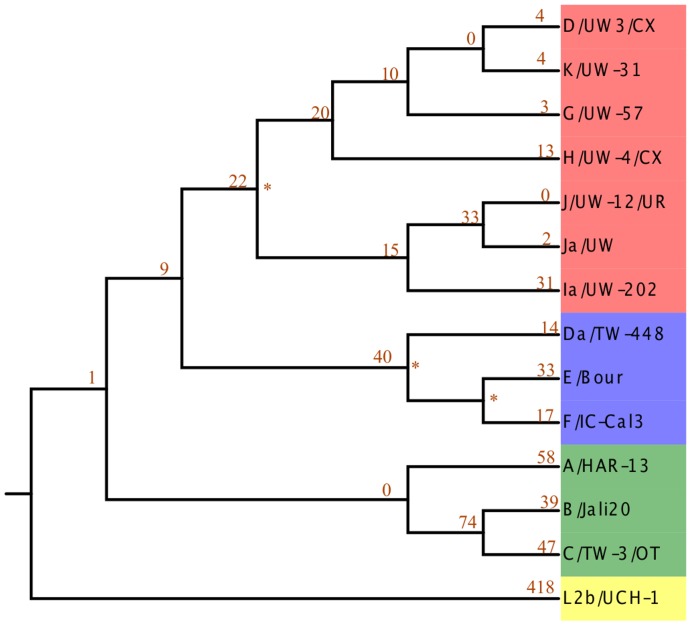
Phylogeny of 100 Tree calculated in the same manner as Figure 2 but instead based on the 100 kb region of the *C. trachomatis* genome selected for targeted amplification. The colors are the same as Figure 3.

In this pilot study, we aimed to capture a 100 kb contiguous section of the *C. trachomatis* genome (genomic locations 100,000–200,000 in the D/UW-3/CX strain [24]; Figure S1 in File S1). This region was chosen because it is outside of the MLST and *ompA* loci and did not contain large repeats. The outline for genotyping the mapped read data was as follows. First, we chose a set of 14 fully sequenced genomes representing diverse known reference strains based on the results of a recent study [7] (Figure 3). We generated SNP pattern files of known SNPs within the 100 kb target region or the whole genome. The reference was the CT_ASR ancestrally reconstructed sequence (see Materials and Methods for details of construction). There were a total of 1,421 and 15,387 SNP positions used, in the targeted 100 kb region and across the entire genome, respectively (Figures 3 and 4). The 100 kb target region contains at least a small number of SNPs unique for each reference strain represented, with the exception of the serotype J strain (J/UW-12/UR), for which there are only 6 representative SNPs in the entire genome. The proportion of variant nucleotides at each known position was identified from the SAM mpileup output. **β** estimates from the linear model that used the binomial probability of observing a SNP at a position were calculated across sites for all 14 references strains. The *binstrain* genotype based on the 100 kb sequenced region of the 14 test genomes were termed here as the “100 kb-genotype”. The genotype based on the whole genomes sequence of the 14 strains, was called the “WG-genotype”.

When challenged with data simulated from the reference genome set, the *binstrain* algorithm accurately matched the MLST-genotype and the proportion of strains present (represented by the estimated β value; Figure 2, Figures S2, S3, and S5 in File S1). Using only the 100 kb target region, we matched the MLST-genotype of strains Ja/UW and D/UW3/CX, which have 2 and 4 strain-unique SNPs respectively (Figure 4). This was despite the fact that the phylogeny of the 100 kb region and the whole genome were not identical (87.5% identity using the R tree.comp tool of the spider package [25]; see Figure 3 and 4) , which showed the robustness of the *binstrain* method. Recent comparative studies have shown that *C. trachomatis* has a history of homologous recombination across its genome [6], [11], [26], [27]. We investigated how this would affect *binstrain* prediction by including 6 *C trachomatis* simulated genome sequences from outside the reference set where there was evidence of recent homologous recombination of DNA from a distantly related lineage. With these strains, we used the whole genome as reference rather than just the 100 kb target in order to maximize the chances of detecting a novel event. The MAP plots were suggestive of simplexes but from the binstrain analysis we observed multiple β values >0.05, indicating that they contained mixtures of SNPs from the reference strains (Figure 2; Figures S7 and S8 in File S1). Therefore, *C. trachomatis* strains likely to contain recent recombination regions produced *binstrain* patterns that reflected their mixed ancestry.

### Development of a PCR microdroplet method for enriching *C. trachomatis* DNA from clinical samples for direct genomic sequencing

**Figure 5:**
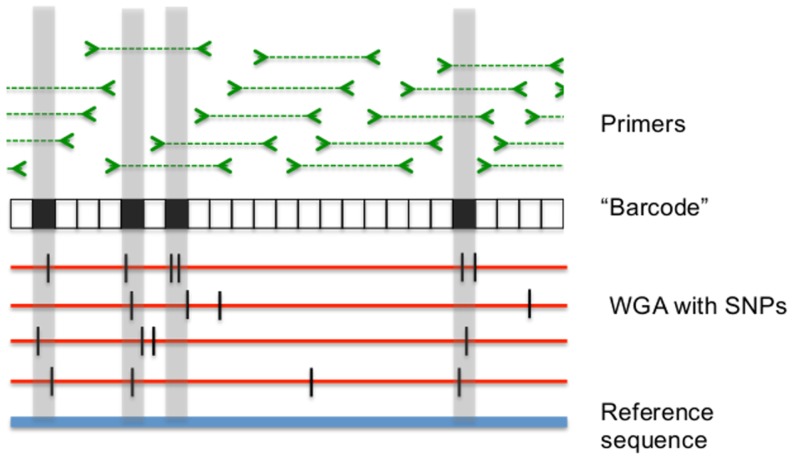
Primer design strategy. Diverse *C. trachomatis* genomes were aligned using Whole Genome Alignment (WGA) software against the *C. trachomatis D/UW3/CX* reference sequence and SNPs (hash lines) were identified (a small number of indels were also identified but were omitted from the figure to make it simpler). The genome was divided into 100 bp blocks. Blocks with a threshold of two or more SNPs are labeled in black and correspond to gray regions in the genome). Starts and ends for primers (amplicon regions in dashed lines) were designed to avoid variable blocks (the black portions of the barcode). Primers were designed to allow approximately 5-fold overlapping amplicon coverage.

We developed a method to enrich, sequence and analyze a 100 kb region of the *C. trachomatis* genome from DNA that had been directly purified from clinical samples. Five hundred primer pairs (primer length = 21 bp) were designed to produce 1.1–1.3. kb overlapping amplicons, giving an average redundancy of amplicon coverage of approximately 5-fold (Table S3 in File S1). The primer pair design was based on the D/UW-3/CX reference sequence (Figure 5). In order to optimize design of the primers, we developed a bioinformatic pathway based on comparative analysis of 12 complete *C. trachomatis* genomes [5]–[11] (Table S1 in File S1). Using the positions of called SNPs and indels from a whole genome alignment by MAUVE [11], [28], we divided the 100 kb region into 100 bp sections assigning a binary code to each; “1” for those containing two or more SNPs, “0” for sections with one or zero SNPs. We used this information to develop an algorithm to optimize the choice of regions for primer design looking for binary strings of 11–13 (i.e., 1100–1300 bp) beginning and ending with “0”. The sequence constraints were fed into Primer3 [12], [13], [29] software to design oligonucleotides. The primers were tiled at intervals of approximately 200 bp on the reference genome.

Multiplex micro-droplet PCR was performed for enrichment followed by high-redundancy sequencing (HiSeq, Illumina). Detailed descriptions of the data are presented in Figures S9 to S23 in File S1. For the initial Set 1 experiment, we used as template 20 ng of gDNA purified from diverse C. *trachomatis* genetic backgrounds (Table 1). The coverage of the 100 kb region ranged from 850× to 7351× (Table 1). Most of this variation in coverage was likely due to Illumina HiSeq multiplexing. Sequence amplification was highly specific; more than 80% of the 100 nucleotide reads aligned to the *C. trachomatis* D/UW3/CX reference genome (Table 1), with fewer than 1% of the *C. trachomatis* reads aligning outside the targeted region. Normalized coverage of reads from all samples measured across the entire 100 kb targeted region was distributed normally with 95% within 10,330.76X–10,303.174X of the mean coverage of 10,316.96X. (Table 1; Figures S15–S17 in File S1).

Two samples from set 1 were sheared on a Covaris acoustic focusing instrument prior to micro-droplet amplification to test whether this improved yield or coverage (Table 1). The non-sheared samples had higher coverage than sheared samples, but we observed consistency in the number of true SNPs recovered from sheared and non-sheared samples and identical *binstrain* patterns. Therefore, subsequent samples were omitted from the shearing step prior to micro-droplet amplification.

For each experiment using a single source genomic DNA (Set 1), we compared the SNPs called against the reference D/UW3/CX genome for each strain against the closest expected reference genome. The definition of a SNP was the nucleotide with the highest count at a variant position. There were zero SNPs when the D/UW-3/CX strain sequence data was mapped against itself, as expected. We also recovered all expected SNPs for Samples 1.1 and 1.2 (strains Ja/47nL and E/5s; 100% sensitivity) and 98% sensitivity for Samples 1.3 (C/TW-3/OT) and 1.4 (H/UW-4/CX) and 93% for 1.5 (L_2_c) (Table 2). However, for the strain identified as “Clinical D” we found that the serotype E genome (E/5s) was the closest match. Based on *ompA* genotyping and MLST, this “Clinical D” was found to be a recombinant between strain D and E (File S1).

**Table 2:** The number of SNPs recovered through the RainDance targeted capture methodology for each of the single strain purified *C. trachomatis* samples used in this study that already has a genome sequence available.

Sample Id	Strain Name	No. of “true/expected” SNPs identified by MUMmer	No. of SNPs recovered after targeted amplification and sequencing	No. of SNP positions with 0× coverage	Sensitivity (%)
1.1	Ja/47nL	288	288	0	100%
1.2	E/5s	276	276	0	100%
1.3	C/TW-3/OT	477	461	16	97%
1.4	H/UW-4/CX	130	128	2	98%
1.5	L2C	735	687	48	93%
1.6	L2C (Not Sheared)	735	697	38	94%
1.7	Clinical D*	NA	NA	NA	NA

The true/expected SNPs were identified by performing a reference mapping of the sample reads to their corresponding genome sequence data.

*There is no complete Clinical D reference sequence.

**Figure 6:**
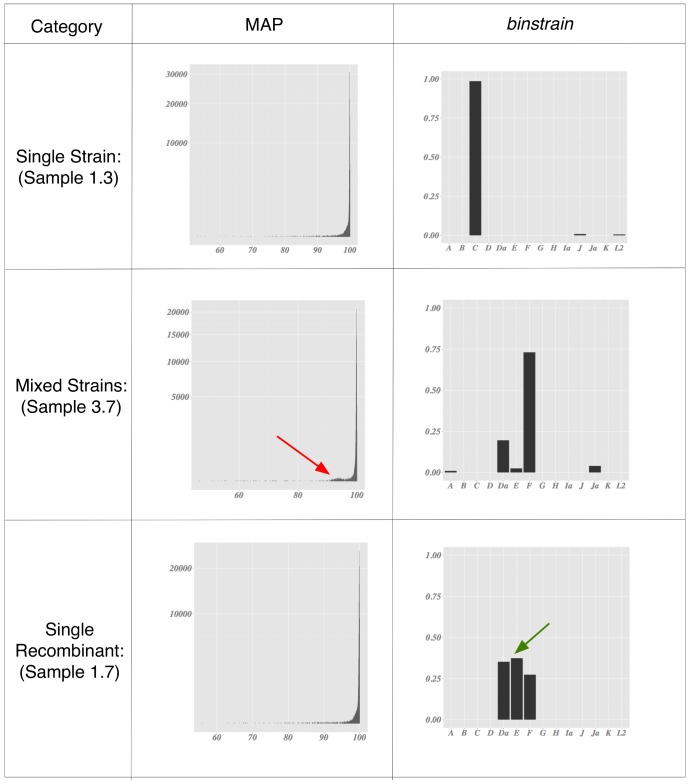
Summary of *binstrain* analysis of selected gDNA and clinical sample data. Format is the same as Figure 2. Note that for a potential mixed infection, we would not be able to currently distinguish between multiple the presence of recombinant and non-recombinant strains, just the proportion of the genotype specific SNPs represented by the **β** values.

*binstrain* analysis on the sequence data from the target region of Set 1 strains produced similar patterns to those seen in the simulated data (Figures 2 and 6; Figures S9 and S10 in File S1). The *binstrain* algorithm successfully retrieved the identity of C/TW-3/OT, H/UW-4/CX and D/UW3/CX with β-values >0.9, indicating that they were close matches to known MLST-genotypes. On the other hand, the “Clinical D” strain *binstrain* pattern (sample 1.7) was an amalgam of 3 *C. trachomatis* reference strains E/Bour (β = 0.37), Da/TW-448 (β = 0.352) and F/IC-Cal3 (β = 0.269). These three strains are all included in Clade 2 of the *C. trachomatis* whole genome phylogeny [7] (Figure 3).

### Sequencing and genotyping *C. trachomatis* directly from clinical sample DNA

We performed two further enrichment experiments on chlamydial gDNA that was purified directly from clinical samples. We chose the remnant NAAT samples because they are the most common clinical sample type available for downstream research studies and normally are just discarded. For Set 2, we extracted DNA from *C. trachomatis* NAAT positive cervical, urethral and urine samples. We did not obtain a sufficient DNA amplicon from micro-droplet amplification of DNA from the two urethral samples but were able to sequence from cervical (4/9) and urine (1/3) sample DNA (Table 1). For Set 3, we used a different methodology for DNA extraction (see Methods) and were able to successfully amplify 8 of 10 (80%) NAAT positive cervical (5/6) and urethral (3/4) samples compared to 5 of 14 (35.7%) samples for Set 2. While the quality of the sequence data from Set 3 was comparable to that derived from the purified gDNA template of Set 1 (Table 1, Figures S15–S17, S21–S23 in File S1), the quality of Set 2 was significantly lower, with the least successful sample (2.4) having only 27.55% reads aligning to the *C. trachomatis* reference (Table 1). Nevertheless, for Set 2, coverage was evenly distributed across the 100 kb reference DNA (Figures S18–S20 in File S1) and we were able to use the data for *binstrain* analysis.

Samples 3.4, 3.5 and 3.7 were mixed infections artificially created by combining purified DNA from two separate clinical samples *ompA*-genotyped Ja and F in proportions of 1∶1, 1∶5 and 1∶50, respectively. The mixture was detected by *binstrain* analysis, with Ja being the dominant β value in 3.4 and F dominant in 3.7, while 3.5 values are intermediate (Figure S13 in File S1). The MAP plots also reveal minor peaks suggestive of mixed culture in 3.4, 3.5 and 3.7 (Figure 6; Figure S14 in File S1). Aside from these samples, all other samples from Sets 2 and 3 appeared to be simplex by visual inspection of MAP plots. However, *binstrain* analysis revealed that most strains contained significant proportion of SNPs mapping to mixes of different serotypes. Only Sample 2.7 contained SNPs mapping to a single reference strain with a β>0.8 (strain E) (Figure S11 in File S1). When we looked in detail at the SNP patterns of these samples 3.18 and 3.19 (Figures S24 and S25 in File S1) we saw evidence for recombination. Samples 3.18 and 3.19 contained all the SNPs common to Clade 2 (Figure 4); however, each also contained SNPs assigned as unique to more than one genome in our SNP matrix of 14 representative strains. Further, the genome-unique SNPs were arranged in contiguous blocks. These patterns suggested localized DNA exchanges (recombination) between strains from different lineages. The MLST profile for samples 2.3, 2.4, 2.10, 2.14 and 3.19 was different from the serotype suggested by the *binstrain* major β values (Table 1). In each of these four cases, there was evidence of recombination in the 100 kb amplified target region.

## Discussion

In this study, we demonstrated PCR amplification based enrichment and sequencing of a 100 Mb portion of the *C. trachomatis* chromosome using as template both purified gDNA, and also DNA extracted directly from remnant NAAT clinical samples without the need for culture. The method we describe is one of several possible approaches to direct sequencing, each with their own advantages and disadvantages. PCR amplification based technology such as RainDance enriches the target sample prior to sequence library construction, compared to sequence capture approaches that use hybridization to enrich existing libraries [30]–[32]. PCR amplification may be an advantage when the target is only a small proportion of the DNA present in the original sample. Using overlapping amplicons we were also able to generate even representation of the target genome sequence. This was evidenced by the fact that *binstrain* analysis on our experimentally amplified and sequenced strains matched results from sequence generated by a random *in silico* simulation. A disadvantage of using PCR amplification is that analysis is limited to already known *C. trachomatis* sequences. Compared to the methods based on direct pull down of *C. trachomatis* cells [12], [13], all sequence capture/amplification approaches require upfront investment in synthesizing large primer libraries, although these costs can be mitigated to an extent by the labor saved in high-throughput sample processing. *C. trachomatis* is an ideal pathogen for development of PCR based enrichment because the genome is small (1.1 Mb), has relatively few repeats and there is low nucleotide sequence diversity between strains [7]. The approach used here could in principal be used for other bacterial species, particularly pathogens that have typically less than 1% DNA sequence diversity in the core genome and limited recent history of limited horizontal gene transfer. This group includes *Mycobacterium tuberculosis*, *Bacillus anthracis* and *Yersinia pestis* [33].

We achieved high quality, even sequence read coverage across the portion of the genome chosen for analysis. However, the methodology can be refined further. Experimental work is necessary to understand the limits of detection in terms of the concentration of the *C. trachomatis* gDNA present and also the concentration of potentially interfering template such as human DNA. Micro-droplet amplification (and other PCR based enrichment technologies) can be extended to capture the whole 1.1 Mb *C. trachomatis* genome at the level of primer coverage used here. Using the whole genome would provide greater sensitivity to detect recombinants, and mixed infections.

We developed a program (*binstrain*) that used a binomial mixture model to decompose the SNPs detected in comparison with a set of representative *C. trachomatis* genomes (Figure 3; Table S1 in File S1). The primary advantage of *binstrain* is that it can report complex information about the admixture of SNPs across a single genome region without the need for full-scale comparative analysis. Additionally, by including SNPs from a larger DNA region (potentially the whole genome), there is more sensitivity to detect strain differences compared to MLST or *ompA* typing. A disadvantage of the *binstrain* algorithm is that it is limited to typing strains within the lineages covered by the SNP matrix. Another issue, as we demonstrated here, is that *binstrain* genotyping can be confounded by strains that contain DNA recently acquired from a distantly related lineage. In order to address this issue, the algorithm could be extended to screen for localized blocks of genotypic divergence. Another useful property would be to identify and report novel mutations and indels not included in the input SNP matrix. Of course, in many cases, two or more *C. trachomatis* strains may be present in the same clinical sample. We showed using both simulated genomes and sequence data from clinical samples that is it possible to identify *C. trachomatis* 100 kb-genotypes and WG-genotypes present in complex mixtures. We used visualization of MAP patterns to detect potential mixed strains in a sample and *binstrain* to predict the distribution of 100 kb-genotype specific signal. The results from this study further support the well-established evidence for recombination among *C. trachomatis* clinical strains [6], [11], [26], [27].

The *binstrain* software provides a framework for a genotyping scheme using whole genome sequences. For this proof-of-concept study, we used as input to *binstrain*, a SNP matrix derived from comparative analysis of 14 genetically diverse reference genomes. Future efforts to sequence the natural diversity of the species will improve how we partition and label SNPs for input into the program. Concentrating on SNPs in the core “clonal frame” portion of the genome, outside of recombination hotspots such as the *ompA* gene, should improve clarity of WG-genotype assignment [6]–[8]. We recently predicted that recombining SNPs in *C. trachomatis* fall into 4 ancestral groups using the STRUCTURE program [7], [34]. The relative proportions of each group of SNPs across the whole genome could be used to produce more robust genotyping signal. As our knowledge of *C. trachomatis* population genomics increases, it may be possible to group SNPs with known geographical or ecological association and/or by their ecological localization (for instance tissue tropism).

It has not been possible until recently to sequence the diversity of uncultured *C. trachomatis* because of the low abundance of the organism, especially in relation to other organisms in the same clinical niche. In addition, more than 95% of the DNA isolated at the mucosal surfaces that the pathogen infects is host–derived [15], [35], and this can swamp low-abundance signals in whole genome shotgun sampling. Even though we did not see naturally mixed infections in this study, the *C. trachomatis* mixed infection rate in STI populations has been estimated at between 2–35% [36]–[39]. These estimates are somewhat preliminary because mixed infections are rarely looked for in the clinical setting. Clinics rely on NAATs for *C. trachomatis* detection and diagnosis but these tests do not provide information on within-species genetic diversity or genotype. Mixed infections are the necessary precursor to homologous recombination. The methodology presented in this work is a step towards better detection of mixed infections and high resolution mapping of regions of DNA exchange within the host. This knowledge could be valuable for assessing the importance of recombination in generating new *C. trachomatis* virulence modalities.

### Data availability

All sequence data was submitted to the National Center for Biotechnology Information Short Read Archive database as Bioproject accession PRJNA225791.

## Supporting Information

File S1 **This file contains text with detailed descriptions of the experiments, MAP** ***binstrain*** **and coverage plots and tables with additional information about synthetic data files.** File S1 also contains the following figures and tables: **Figure S1**, Map of The *C. trachomatis* D/UW3/CX genome. **Figure S2**, *binstrain* β estimates for the single strain/uni-mixture entire (whole) genome simulated samples and *binstrain* beta estimates for the single strain/uni-mixture 100 kb targetedsimulated samples. **Figure S3**, *binstrain* β estimates for the 10 bi-mixture entire (whole) genome simulated samples and *binstrain* beta estimates for the 10 bi-mixture 100 kb targeted simulated samples. **Figure S4**, MAP plots for the whole genome simulated 10 bi-mixture samples and100 kb targeted simulated 10 bi-mixture samples. **Figure S5**, *binstrain* β estimates for the 4 tri mixture entire (whole) genome simulated samples and *binstrain* β estimates for the 4 tri mixture 100 kb targeted simulated samples. **Figure S6**, MAP plots for the whole genome simulated 4 tri-mixture samples and 100 kb targeted simulated 10 tri-mixture samples. **Figure S7**, *binstrain* β estimates for 6 simulated recombinant strains. **Figure S8**, MAP plots for the whole genome simulated recombinant samples. **Figure S9**, *binstrain* β estimates for experimental Set 1. **Figure S10**, MAP plots for the 100 kb regions of Set1. **Figure S11**, *binstrain* β estimates for clinical sample Set 2. **Figure S12**, MAP plots for the real 100 kb regions of Set 2. **Figure S13**, *binstrain* β estimates for clinical sample Set 3. **Figure S14**, MAP plots for the 100 kb targeted genome clinical samples of Set 3. **Figure S15**, Distribution of the Normalized Average Coverage across the entire 100 kb targeted region in Set 1. **Figure S16**, Normalized standard deviation of coverage in Set 1. **Figure S17**, Box plots representing the distribution of the normalized coverage in bins of 10 kB regions across the entire 100 kb region targeted in sample set 1. **Figure S18**, Distribution of the Normalized Average Coverage across the entire 100 kb targeted region of the clinical samples in Set 2. **Figure S19**, Normalized standard deviation of coverage of sample Set 3. **Figure S20**, Box plots representing the distribution of the normalized coverage in bins of 10 kB regions across the entire 100 kb region targeted in sample set 2. **Figure S21**, Distribution of the Normalized Average Coverage across the entire 100 kb targeted region of the clinical samples in Set 3. **Figure S22**, Normalized standard deviation of coverage of samples in Set 3. **Figure S23**, Box plots representing the distribution of the normalized coverage in bins of 10 kB regions across the entire 100 kb region targeted in sample set 3. **Figure S24**, Breakdown of *binstrain* results for sample 3.18. **Figure S25**, Breakdown of *binstrain* results for sample 3.19. **Table S1**, List of *C. trachomatis* genomes used for primer design, ancestral sequence regeneration and whole genome MAUVE alignment to generate the SNP pattern file used in this study. **Table S2**, List of the *C. trachomatis* genomes used for simulating uni, bi and tri artificial mixed infected samples and their *binstrain* beta estimates.
(PDF)
