# Supplementary material for: Direct Amplification, Sequencing and Profiling of Chlamydia trachomatis Strains in Single and Mixed Infection Clinical Samples
Source: PLoS One. 2014 Jun 27;9(6):e99290. doi: 10.1371/journal.pone.0099290 (PMC4074039; doi:10.1371/journal.pone.0099290)
Supplement: File S1 — This file contains text with detailed descriptions of the experiments, MAP binstrain and coverage plots and tables with additional information about synthetic data files. File S1 also contains the following figures and tables: Figure S1, Map of The C. trachomatis D/UW3/CX genome. Figure S2, binstrain β estimates for the single strain/uni-mixture entire (whole) genome simulated samples and binstrain beta estimates for the single strain/uni-mixture 100 kb targetedsimulated samples. Figure S3, binstrain β estimates for the 10 bi-mixture entire (whole) genome simulated samples and binstrain beta estimates for the 10 bi-mixture 100 kb targeted simulated samples. Figure S4, MAP plots for the whole genome simulated 10 bi-mixture samples and100 kb targeted simulated 10 bi-mixture samples. Figure S5, binstrain β estimates for the 4 tri mixture entire (whole) genome simulated samples and binstrain β estimates for the 4 tri mixture 100 kb targeted simulated samples. Figure S6, MAP plots for the whole genome simulated 4 tri-mixture samples and 100 kb targeted simulated 10 tri-mixture samples. Figure S7, binstrain β estimates for 6 simulated recombinant strains. Figure S8, MAP plots for the whole genome simulated recombinant samples. Figure S9, binstrain β estimates for experimental Set 1. Figure S10, MAP plots for the 100 kb regions of Set1. Figure S11, binstrain β estimates for clinical sample Set 2. Figure S12, MAP plots for the real 100 kb regions of Set 2. Figure S13, binstrain β estimates for clinical sample Set 3. Figure S14, MAP plots for the 100 kb targeted genome clinical samples of Set 3. Figure S15, Distribution of the Normalized Average Coverage across the entire 100 kb targeted region in Set 1. Figure S16, Normalized standard deviation of coverage in Set 1. Figure S17, Box plots representing the distribution of the normalized coverage in bins of 10 kB regions across the entire 100 kb region targeted in sample set 1. Figure S18, Distribution of the Normalized Averag [file pone.0099290.s001.pdf]

Supplemental Text, Tables and Figures.

Ascertainment of *C. trachomatis* genomic strain type using a binomial mixture model

In order to target capture experiments, we developed a binomial mixed model (*binstrain*) to predict the most likely genetic background(s) of the *C. trachomatis* strain. The advantages of the model included its execution speed, ability to update predictions with new reference genome data and, critically, the ability to identify mixed infections. The model is described in detail in the Materials and Methods. As input for *binstrain*, we created matrices used to assign the sequence data at serovar-level using data from 14 *C. trachomatis* reference genome projects. For each experiment, the mpileup file of the reads mapped against the reference target 100kb region and /or on the entire CT_ASR genome was queried at each of these positions and the binomial coefficient of probability was calculated across sites for all 14 serovar references.

***binstrain* analysis of simulated data**

We first tested the ability of *binstrain* algorithm to identify 1) single strain samples, 2) mixed infections with 2 to 3 strains present and 3) recombinant strains using simulated sequence data. Artificial FASTQ files with various proportions of coverage were generated for the 13 *C. trachomatis* reference strains and 6 recombinant strains (Supplementary Table S1). Altogether there were 5 single strain/uni-mixture, 10 bi-mixture, 4 tri-mixture samples and 6 recombinant simulated samples. Two or three such artificial FASTQ files were merged to create the bi and tri mixture samples. The analysis was performed using both the simulated datasets from the 100kb targeted region (except for the recombinant strains where whole genome simulation was conducted) as well as targeting the entire genome of *C. trachomatis.*

For the simulated single strain samples, the *binstrain* algorithm accurately predicted the presence of the simulated reference strain in each sample with $\beta \geq 0.94$. Supplementary figures S2 (a) and (b) shows the estimated β values of all the 5 single strain simulated samples generated for the whole genome and the targeted 100kb regions respectively. The predicted β values from the 10 bi mixture samples for the whole genome and targeted 100kb simulated samples are shown in Supplementary

Figure S3. All the estimated β values were highly correlated to the reference *C trachomatis* strains artificially mixed in each of the 10 bi mixture samples. Moreover, the predicted β values were highly correlated to the proportions (coverage used in the simulation) of each of the 2 reference strains present in each of the bi mixture except for the bi mixture sample 6 (E/Bour (3000X) + F/IC-Cal3 (5500X)). The MAP plots for the 10 bi-mixture samples are shown in Supplementary Figure S4. For the bi mixture sample 6, *binstrain* predicted the presence of E/Bour (β=0.501) with a higher β value than that for F/IC-Cal3 (β=0.481) when the actual proportion of the latter strain was higher than the former (35.30:64.70). This might be because of the close proximity E and F strains (Figures 2 and 3) where the high number of shared SNP positions may make discrimination less sensitive. In the four simulated tri- mixtures (Supplementary figure S5), *binstrain* accurately estimated the composition of the strains present in each of the samples. The MAP plots for the 4 tri-mixture samples are shown in Supplementary Figure S6.

Comparative genomic evidence has shown that there is a history of homologous recombination in *C. trachomatis*. We also investigated how this would affect *binstrain* prediction by simulating 6 *C. trachomatis* genome sequences where we have detected recent import of DNA from a distantly related lineage. The estimated β values are shown in Supplementary figure S7 and the MAP plots in Supplementary figure S8. The D/2s strain was identified to be recombinant between D and Ia serovars using *ompA* genotyping/MLST. Our analysis using *binstrain* assigned D/2s sample as a single strain sample with the highest estimated β values for Ia/UW-202 reference strain (β=0.968). There were genetic contributions of D/UW-3/CX. This was probably because homologous recombination from D/UW-3/CX might have happened only to a very small region of the Ia/UW-202 chromosome. Similarly D/43nl was inferred as a recombinant strain between D and G serovars and our predictions assigned the D/43nl whole genome sample as highly similar to D/UW-3/CX strain (β=0.863), The strain H/18s was previously inferred as recombinant strain between H and G serovars. This is reflected in the betas for the whole genome simulated sample of H/18s with H/UW-4/CX (β=0.446) and G/UW-57 (β=0.335) along with Ia/UW-202 (β=0.134). We also simulated the targeted whole genome data for the recently identified LGV strain L2C, which was identified as an amalgam of both L2 and D serovars (with a significantly large DNA import from D/UW-3/CX). Our *binstrain* analysis predicted the presence of L2

reference strain with the highest estimated β (β=0.921) and the second highest β value was for D/UW-3/CX (β=0.03). These results suggested that *binstrain* algorithm is sensitive enough to identify the genotypes present in a recombinant strain provided there was a significant proportion of the genome imported.

Composition of samples in Set 1

In each case for Set 1, except for Clinical D we have a completely sequenced genome for comparison. The *binstrain* algorithm successfully retrieved the identity of C/TW-3/OT, H/UW-4/CX and D/UW3/CX with very high estimated values, indicating that they were close matches to known genotypes (Supplementary Data S12). For the "Clinical D" (samples 1.7,1.8) strain, *binstrain* pattern was an amalgam of 3 *C. trachomatis reference strains* E/Bour ($\beta = 0.378$), Da/TW-448 ($\beta = 0.352$) and F/IC-Cal3 ($\beta = 0.269$) (supplementary figure S9). These 3 strains are phylogenetically close and are included in the Clade 2 of the *C. trachomatis* whole genome phylogeny [1]. Clinical D was previously assigned the genotype of D by *ompA* genotyping and as E using MLST. The L2C samples (1.5,1.6) were correctly assigned to the L2 serovar with the highest estimated β value of 0.986. When we used whole genome simulated RainDance sample data (see above), the β value for L2 was 0.921 along with the second highest β value for D/UW-3/CX confirming that L2C as a recombinant LGV strain with DNA import from D/UW-3/CX (Figure S7). Experimental results from the 100kb target Ja/47nL sample 1.2 had estimated values for strains F/IC-Cal3 (β =0.794) and Da/TW-448 (β=0.205), indicating the possibility of a mixed infection or a recombinant strain (Supplementary Figure S9). With simulated whole genome data of Ja/47nl we obtained matches to F/IC-Cal3 (estimated β value was 0.629) and Da/TW-448 (estimated β value was 0.099), along with presence of E/Bour (estimated β value of 0.218) (Supplementary Figure S7). The Ja/47nl strain was predicted as a Ja serovar by *ompA* genotyping and as E serovar based on MLST. Joseph et al. 2012 [1] suggested that Ja/47nL was an ancestral recombinant with DNA from clade 2 (88%) (that includes all the E and F serovars) strains and a minor proportion from clade 4 (1%) (that includes all the D serovar strains), which is consistent with the prediction of the *binstrain* algorithm here. For the 100kb targeted E/5s sample 1.1, *binstrain* analysis assigned it to F/IC-Cal3 (β =0.757), Da/TW-448 (β=0.145) and E/Bour (β=0.07) (Supplementary Figure S9), while the

simulated *binstrain* analysis of the entire genome of E/5s predicted increased proportion of E/Bour (β=0.280) along with the presence of F/IC-Cal3 (β=0.620) and Da/TW-448 (β=0.082) (Supplementary Figure S6). The E/5s strain was predicted as an E serovar by *ompA* genotyping and as Ja serovar based on MLST analysis (Table 1). Our previous genomic analysis determined E/5s strain had a higher proportion of ancestral genotype source from Clade 2 (95%) and a lower proportion from clade 4 (0.05), that, which is consistent with the *binstrain* analysis. The MAP plots for the Set 1 samples are shown in Supplementary Figure S10. Since all were single strain cultures, we saw no evidence of mixture, as expected.

Composition of clinical samples in Set 2

Using *binstrain* we identified sample 2.3 as containing predominately Ia (β=0.836) and J (β=0.163) SNPs, even though *ompA* genotyping and MLST indicated that the strain was a K (Supplementary Figure S11; Table 1). This suggested a recombinant genome. Sample 2.4, was typed aa putative recombinant E and Da, by MLST/ *ompA*. Based on the 100 kb target region, *binstrain* indicated the sample could be a mixed infection with the presence of L2, E and A serovars. Sample 2.10, an E by ompA genotyping and Da by MLST (Table 1), had the highest estimate for the *C. trachomatis* F/IC-Cal3 reference strain (β=0.692) along with traces of Da/TW-448 (β=0.172), E/Bour (β=0.114) and A/HAR-13 (β=0.020) indicating this sample could be either a mixed infection or a F serovar strain with recombination (Supplementary Figure S11). Sample 2.7, an E by both *ompA* and MLST, was also predicted by *binstrain* algorithm as a single infection sample with the highest estimated β value of 0.834 for the E/Bour reference strain. Sample 2.14 was also predicted either as a mixed infection or a recombinant F strain with F/IC-Cal3 (highest proportion, β=0.766), along with Da/TW-448 (β=0.218) and traces of E/Bour (β=0.014) (Figure S11). The MAP plots for the Set 2 samples are shown in Supplementary Figure S12.

Composition of clinical samples in Set 3

The results for the statistical modeling using the *binstrain* algorithm for identifying the underlying strains responsible for the infection for each of the clinical samples in Set 3 is shown in

Supplementary Figure S13. Samples 3.4, 3.5 and 3.7 were clinical mixed samples of 2 serovars Ja and F identified by ompA/MLST genotyping and mixed in varying proportion (Table 1). Sample 3.4 contained equal proportions of Ja and F and *binstrain* predicted the presence of Ja and F but the β values were not exactly proportional to the ratios of the 2 serovars mixed (Supplementary Figure S13). Sample 3.5 contained Ja and F in 1:5 ratio and binstrain predicted the highest β value for F/IC-Cal3 (β=0.492) and the second highest β value was for Ja/UW (β=0.362), which was proportional to the quantities of clinical Ja and F samples mixed (Supplementary Figure S13). Similarly, sample 3.7 had 1:50 ratio of Ja:F clinical serovars and binstrain accurately predicted the presence of F/IC-Cal3 with the highest β value (β=0.730) and Ja/UW with a β of 0.0397, which was proportional to the content of strains present in that mixed infection (Supplementary Figure S13; Table 1). Clinical samples 2.16 and 3.18 were initially genotyped respectively as F and E serovars by both *ompA* and MLST analysis (Table 1). *binstrain* predicted the presence of F/IC-Cal3 (β=0.766), Da/TW-448 (β=0.213) along with traces of E/Bour (β=0.014) in sample 16. For sample 3.18, *binstrain* estimated β values for F/IC-Cal3 (β=0.497), Da/TW-448 (β=0.255) and E/Bour (β=0.238). Similarly sample 3.19 was initially genotyped as D by *ompA* genotyping and E as MLST and *binstrain* assigned it to F/IC-Cal3 and Da/TW-448 with β values 0.802 and 0.196 respectively. Sample 3.15 and 3.17 were initially genotyped as Ia serovar and our analysis also predicted the presence of Ia/UW-202 along with traces of J/UW-12/UR for both the samples (Supplementary Figure S13; Table 1). Both Ia/UW-202 and J/UW-12/UR are phylogenetically close and forms a sub-clade within Clade 4 of the *C. trachomatis* phylogeny. The MAP plots for the Set 3 samples are shown in figure Supplementary Supplementary Figure S14. The minor subpopulations present in mixed strains 3.4, 3.5 and 3.7 can be clearly seen.

Supplementary Figure Legends

Supplementary Figure S1. Map of The *C. trachomatis* D/UW3/CX genome [2] showing the locations of the 100kb amplified target region, *ompA*, and the 7 MLST loci (*lysS*, *yhgB*, *glyA*, *mdhC*, *pykF*, *pdhA*, *leuS*) [3]. For reference, the 2 rRNA operons are shown in pink.

Supplementary Figure S2. a) *binstrain* β estimates for the single strain/uni-mixture entire (whole) genome simulated samples. b) *binstrain* beta estimates for the single strain/uni-mixture 100kb targeted simulated samples. Estimated β's represents the proportion of the presence of *C. trachomatis* strains present/responsible for the infection in the simulated sample.

Supplementary Figure S3. a) *binstrain* beta estimates for the 10 bi-mixture entire (whole) genome simulated samples. b*) binstrain* beta estimates for the 10 bi-mixture 100kb targeted simulated samples. Estimated β's represents the proportion of the presence of *C. trachomatis* strains present/responsible for the infection in the simulated sample.

Supplementary Figure S4 a) The MAP plots for the whole genome simulated 10 bi-mixture samples. b) The MAP plots for the 100kb targeted simulated 10 bi-mixture samples. In order to detect mixed strain cultures, we plotted a statistic we termed 'Major Allele Percentage' (MAP), defined as the percentage of the most common nucleotide at each position of the sequence read mpileup table (with a arbitrary minimum cutoff of considering only samples of at least 100x coverage redundancy).

Supplementary Figure S5 a) *binstrain* beta estimates for the 4 tri mixture entire (whole) genome simulated samples. b) *binstrain* beta estimates for the 4 tri mixture 100kb targeted simulated samples. Estimated β's represents the proportion of the presence of *C. trachomatis* strains present/responsible for the infection in the simulated sample.

Supplementary Figure S6 a) The MAP plots for the whole genome simulated 4 tri-mixture samples. b) The MAP plots for the 100kb targeted simulated 10 tri-mixture samples. In order to detect mixed strain cultures, we plotted a statistic we termed 'Major Allele Percentage' (MAP), defined as the percentage of the most common nucleotide at each position of the sequence read mpileup table (with a arbitrary minimum cutoff of considering only samples of at least 100x coverage redundancy).

Supplementary Figure S7. *binstrain* beta estimates for the 6 recombinant strain. The entire (whole) genome of each recombinant strain was simulated samples. Estimated β's represents the proportion of the presence of *C. trachomatis* strains present/responsible for the infection in the simulated sample.

Supplementary Figure S8. The MAP plots for the whole genome simulated 6 recombinant samples. In order to detect mixed strain cultures, we plotted a statistic we termed 'Major Allele Percentage' (MAP), defined as the percentage of the most common nucleotide at each position of the sequence read mpileup table (with a arbitrary minimum cutoff of considering only samples of at least 100x coverage redundancy).

Supplementary Figure S9. *binstrain* beta estimates of the raindance experiment sample Set 1. Here the raindance samples were generated to target the 100kb region (region between the genomic coordinates of 100,000 and 200,000). Estimated β's represents the proportion of the presence of *C. trachomatis* strains present/responsible for the infection in the purified gDNA sample.

Supplementary Figure S10. The MAP plots for the real 100kb targeted genome samples of Set1. In order to detect mixed strain cultures, we plotted a statistic we termed 'Major Allele Percentage' (MAP), defined as the percentage of the most common nucleotide at each position of the sequence read mpileup table (with a arbitrary minimum cutoff of considering only samples of at least 100x coverage redundancy).

Supplementary Figure S11. *binstrain* β estimates of the raindance experiment clinical sample Set 2. Here the raindance samples were generated to target the 100kb region (region between the genomic coordinates of 100,000 and 200,000) of *C. trachomatis* chromosome. Estimated β's represents the proportion of the presence of *C. trachomatis* reference genomes present/responsible for the infection in the clinical sample.

Supplementary Figure S12. The MAP plots for the real 100kb targeted genome clinical samples of Set2. In order to detect mixed strain cultures, we plotted a statistic we termed 'Major Allele Percentage' (MAP), defined as the percentage of the most common nucleotide at each position of the sequence read mpileup table (with a arbitrary minimum cutoff of considering only samples of at least 100x coverage redundancy).

Supplementary Figure S13. binstrain beta estimates of the Raindance experiment clinical sample Set 3. Here the raindance samples were generated to target the 100kb region (region between the genomic coordinates of 100,000 and 200,000) of C. trachomatis chromosome. Estimated β's represents the proportion of the presence of *C. trachomatis* strains present/responsible for the infection in the clinical sample.

Supplementary Figure S14. The MAP plots for the real 100kb targeted genome clinical samples of Set 3. In order to detect mixed strain cultures, we plotted a statistic we termed 'Major Allele Percentage' (MAP), defined as the percentage of the most common nucleotide at each position of the sequence read mpileup table (with a arbitrary minimum cutoff of considering only samples of at least 100x coverage redundancy).

Supplementary Figure S15. Distribution of the Normalized Average Coverage across the entire 100kb targeted region of samples in Set 1.

Supplementary Figure S16. Normalized standard deviation of coverage of samples in Set 1. After normalization, coverage measured across the entire 100kb targeted region for the Set 1 was found to be distributed normally with an estimated mean coverage of 10,316.96X (95% C. I = 10,330.76X – 10,303.174X).

Supplementary Figure S17. Box plots representing the distribution of the normalized coverage in bins of 10kB regions across the entire 100kb region targeted in sample set 1. All the samples were normalized an average coverage of 10,000X for comparative analysis.

Supplementary Figure S18. Distribution of the Normalized Average Coverage across the entire 100kb targeted region of the clinical samples in Set 2.

Supplementary Figure S19. Normalized standard deviation of coverage of sample Set 3. After normalization, coverage measured across the entire 100kb targeted region for the Set 2 was found to be distributed normally with an estimated mean coverage of 9963.83X (95% C. I = 9993.17 – 9993.17) with a standard deviation of 4734.3 X (95% C. I= 4755.1 – 4713.7).

Supplementary Figure S20. Box plots representing the distribution of the normalized coverage in bins of 10kB regions across the entire 100kb region targeted in sample set 2. All the samples were normalized an average coverage of 10,000X for comparative analysis.

Supplementary Figure S21. Distribution of the Normalized Average Coverage across the entire 100kb targeted region of the clinical samples in Set 3.

Supplementary Figure S22. Normalized standard deviation of coverage of samples in Set 3. After normalization, coverage measured across the entire 100kb targeted region for the Set 3 was found to be distributed normally with an estimated mean coverage of 9962.64X (95% C. I = 9948.622 – 9976.65X) with a standard deviation of 2261.24.

Supplementary Figure S23. Box plots representing the distribution of the normalized coverage in bins of 10kB regions across the entire 100kb region targeted in sample set 3. All the samples were normalized an average coverage of 10,000X for comparative analysis.

Supplementary Figure S24. Breakdown of *binstrain* results for sample 3.18. (a) portion of tree (Fig 2a) showing clade 2 and SNPs with the 100 kb target assigned to branches A-E. (b) *binstrain* results for 3.18 (see Fig S13). (c) Plot for each of the 5 classes of SNP in panel (a) showing the locations of SNPs within the 100kb target (using Ct strain D coordinates). All 40 and 15 SNPs in the A and B classes were represented along with 4/14 Da, 12/33 E and 5/17 F. The height of the bar is proportion to the allele frequency.

Supplementary Figure S25. Breakdown of *binstrain* results for sample 3.19. Same as layout as S21 except that there were no SNPs specific to the serotype E genome. All 40 and 15 SNPs in the A and B classes were represented along with 5/14 Da and 7/17 F.

Supplementary Tables

Supplementary Table S1. List of *C. trachomatis* genomes used for primer design, ancestral sequence regeneration and whole genome MAUVE alignment to generate the SNP pattern file used in this study.

Supplementary Table S2. List of the *C. trachomatis* genomes used for simulating uni, bi and tri artificial mixed infected samples and their *binstrain* beta estimates.

Supplementary Data References

1. Joseph, S. J. & Read, T. D. Genome-wide recombination in Chlamydia trachomatis. *Nat Genet* **44,** 364–366 (2012).
2. Stephens, R. S. *et al.* Genome sequence of an obligate intracellular pathogen of humans: Chlamydia trachomatis. *Science* **282,** 754–759 (1998).
3. Dean, D. *et al.* Predicting phenotype and emerging strains among Chlamydia trachomatis infections. *Emerging Infect Dis* **15,** 1385–1394 (2009).

Figure S1

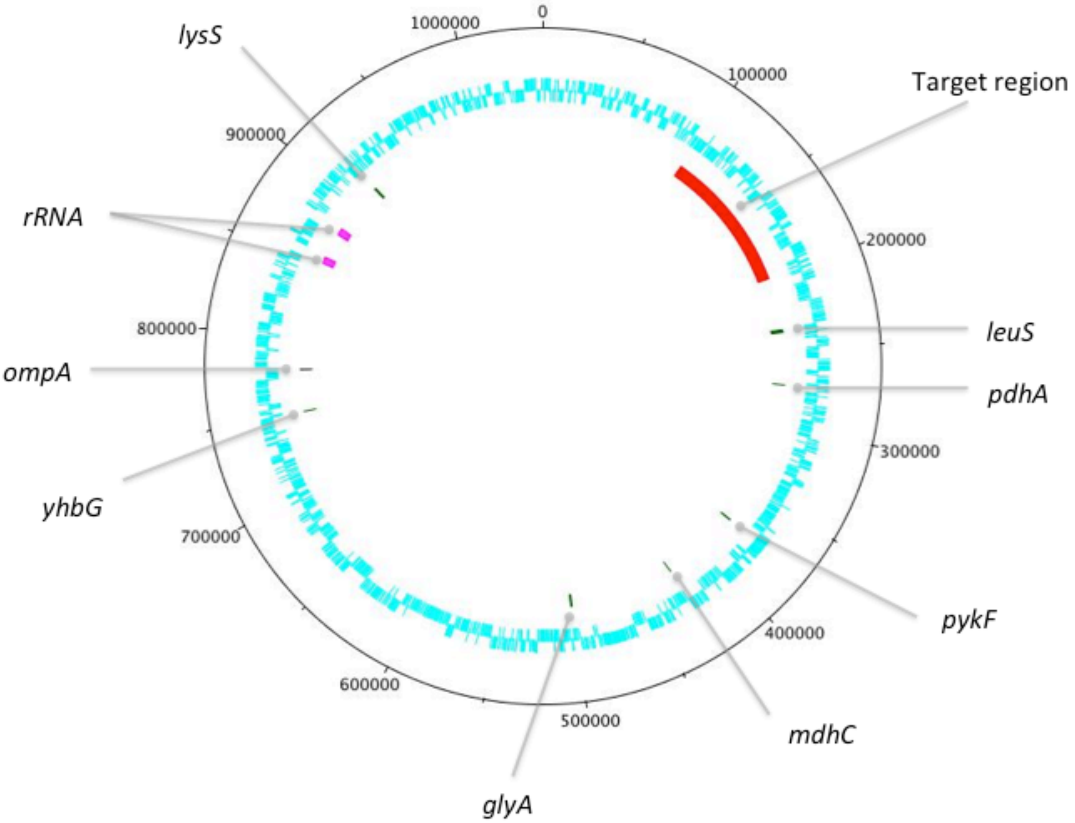

0
1000000
100000
Target region
lysS
900000
rRNA
200000
leuS
800000
ompA
pdhA
300000
yhbG
700000
pykF
400000
600000
500000
mdhC
glyA

Figure S2 (a)

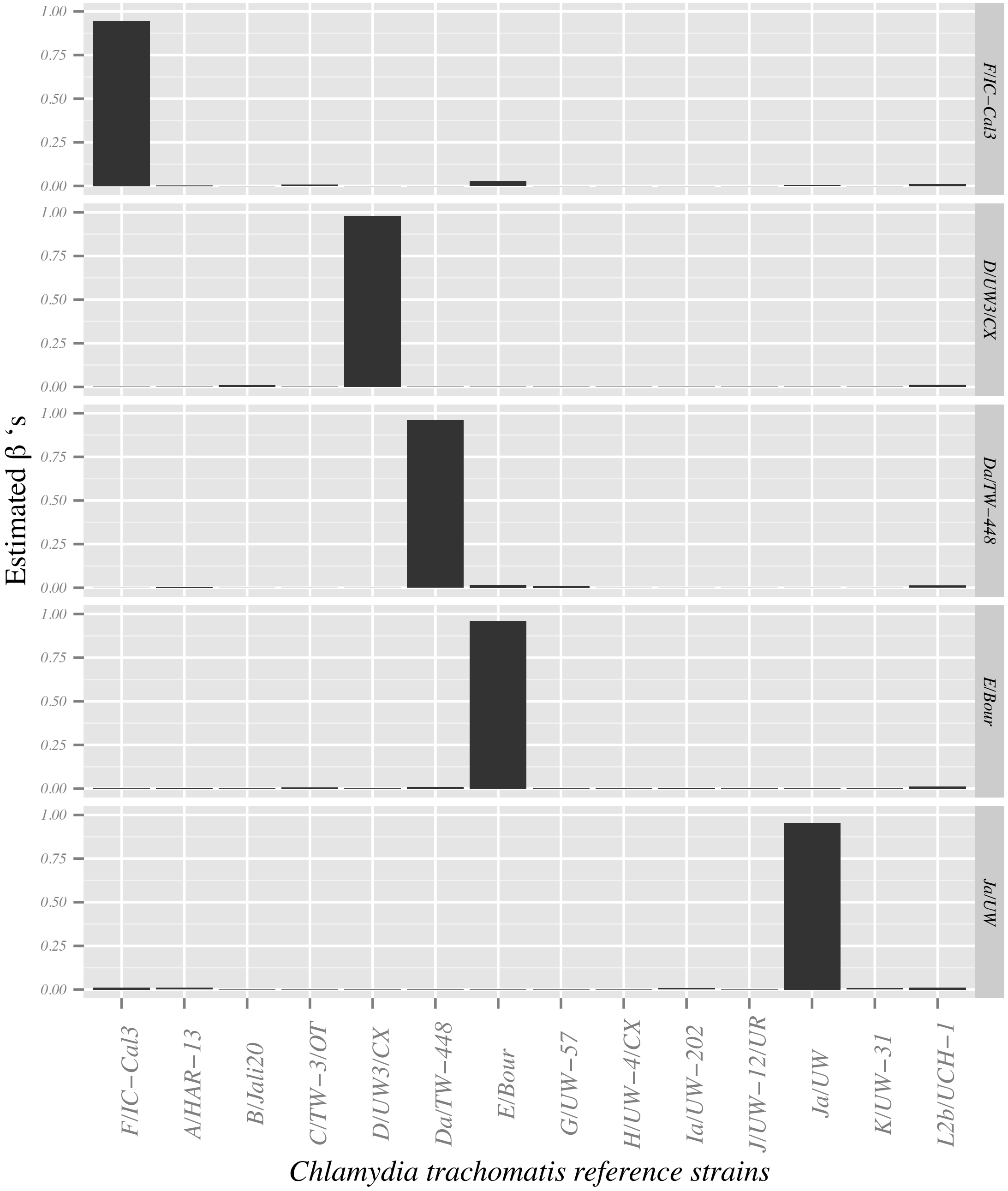

Estimated β 's
1.00
0.75
0.50
0.25
0.00
F/IC−Cal3
D/UW3/CX
Da/TW−448
E/Bour
Ja/UW
F/IC−Cal3
A/HAR−13
B/Jali20
C/TW−3/OT
D/UW3/CX
Da/TW−448
E/Bour
G/UW−57
H/UW−4/CX
Ia/UW−202
J/UW−12/UR
Ja/UW
K/UW−31
L2b/UCH−1
Chlamydia trachomatis reference strains

Figure S2 (b)

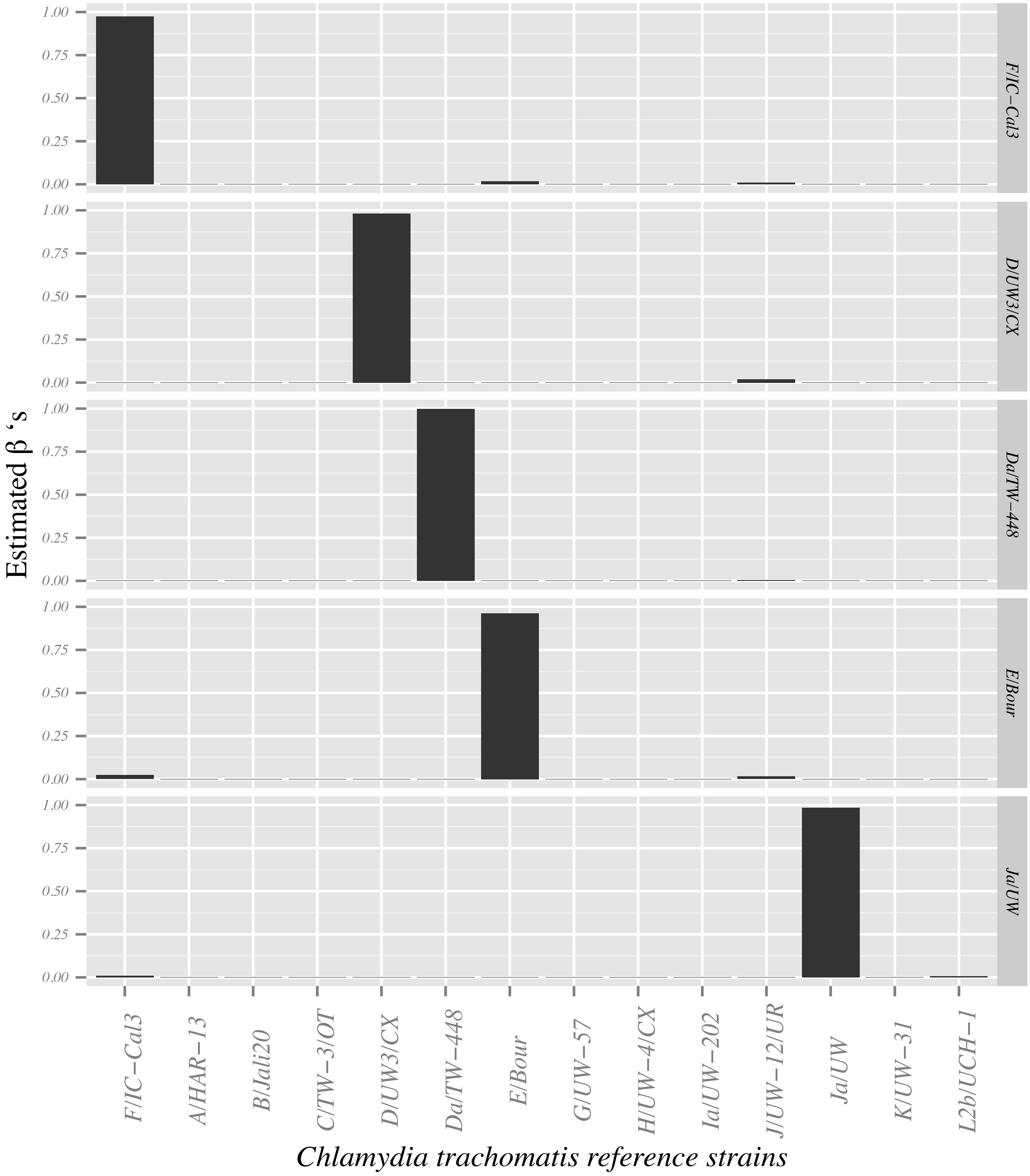

Estimated β 's
1.00
0.75
0.50
0.25
0.00
F/IC−Cal3
D/UW3/CX
Da/TW−448
E/Bour
Ja/UW
F/IC−Cal3
A/HAR−13
B/Jali20
C/TW−3/OT
D/UW3/CX
Da/TW−448
E/Bour
G/UW−57
H/UW−4/CX
Ia/UW−202
J/UW−12/UR
Ja/UW
K/UW−31
L2b/UCH−1
Chlamydia trachomatis reference strains

Figure S3 (a)

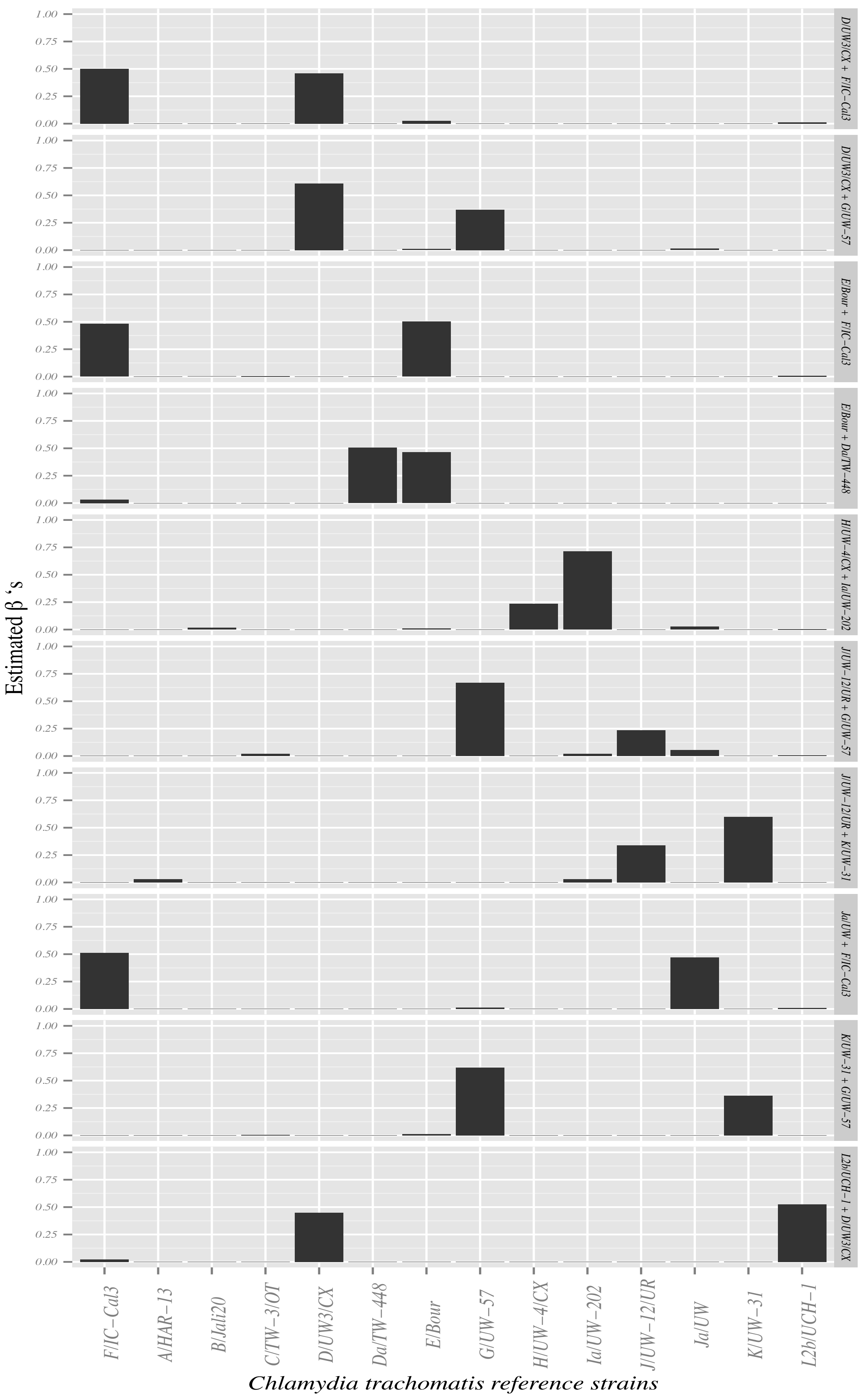

Estimated β 's
1.00
0.75
0.50
0.25
0.00
D/UW3/CX + F/IC−Cal3
D/UW3/CX + G/UW−57
E/Bour + F/IC−Cal3
E/Bour + Da/TW−448
H/UW−4/CX + Ia/UW−202
J/UW−12/UR + G/UW−57
J/UW−12/UR + K/UW−31
Ja/UW + F/IC−Cal3
K/UW−31 + G/UW−57
L2b/UCH−1 + D/UW3/CX
F/IC−Cal3
A/HAR−13
B/Jali20
C/TW−3/OT
D/UW3/CX
Da/TW−448
E/Bour
G/UW−57
H/UW−4/CX
Ia/UW−202
J/UW−12/UR
Ja/UW
K/UW−31
L2b/UCH−1
Chlamydia trachomatis reference strains

Figure S3 (b)

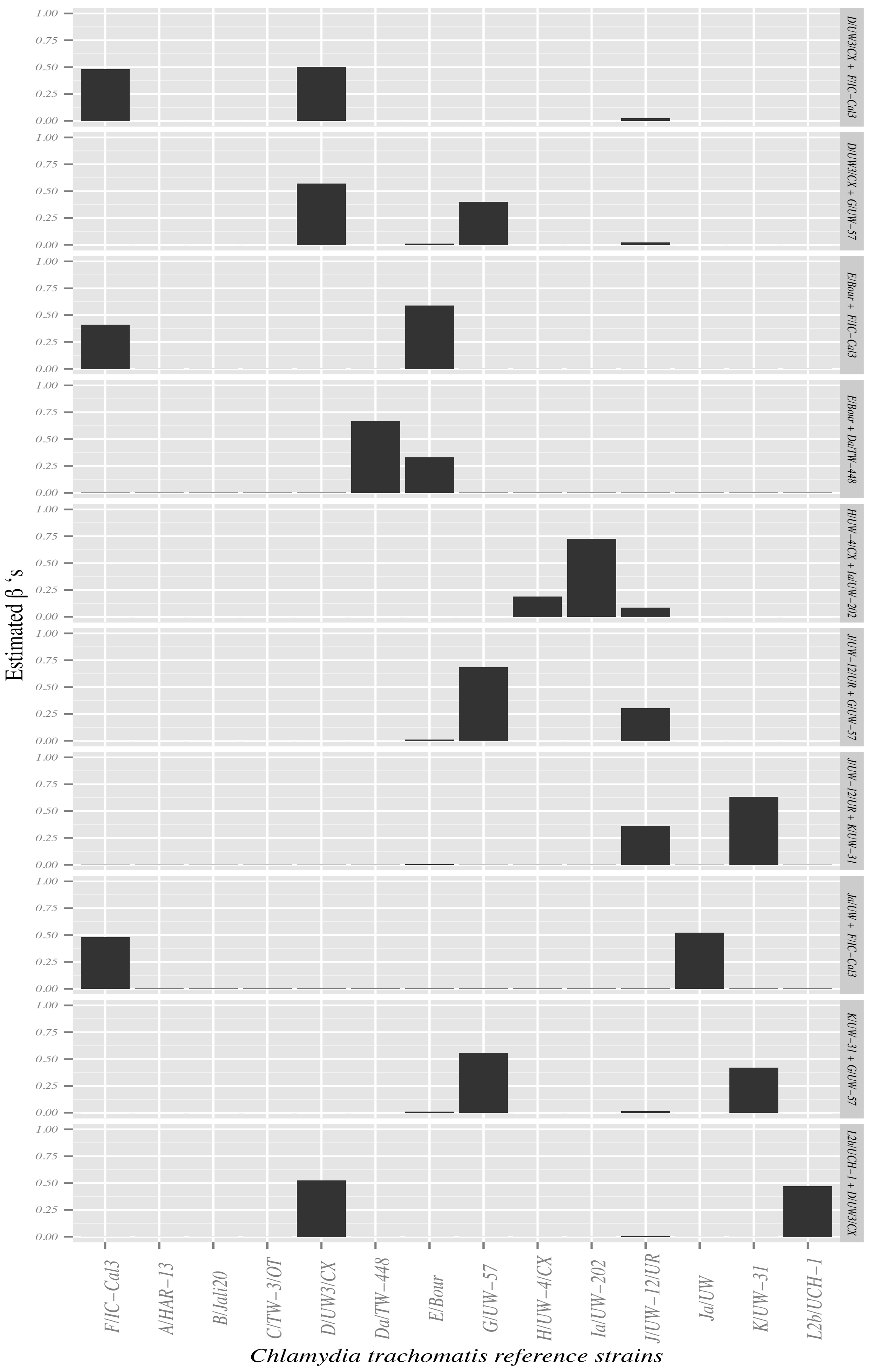

Estimated β 's
1.00
0.75
0.50
0.25
0.00
D/UW3/CX + F/IC−Cal3
D/UW3/CX + G/UW−57
E/Bour + F/IC−Cal3
E/Bour + Da/TW−448
H/UW−4/CX + Ia/UW−202
J/UW−12/UR + G/UW−57
J/UW−12/UR + K/UW−31
Ja/UW + F/IC−Cal3
K/UW−31 + G/UW−57
L2b/UCH−1 + D/UW3/CX
F/IC−Cal3
A/HAR−13
B/Jali20
C/TW−3/OT
D/UW3/CX
Da/TW−448
E/Bour
G/UW−57
H/UW−4/CX
Ia/UW−202
J/UW−12/UR
Ja/UW
K/UW−31
L2b/UCH−1
Chlamydia trachomatis reference strains

Figure S4 (a)

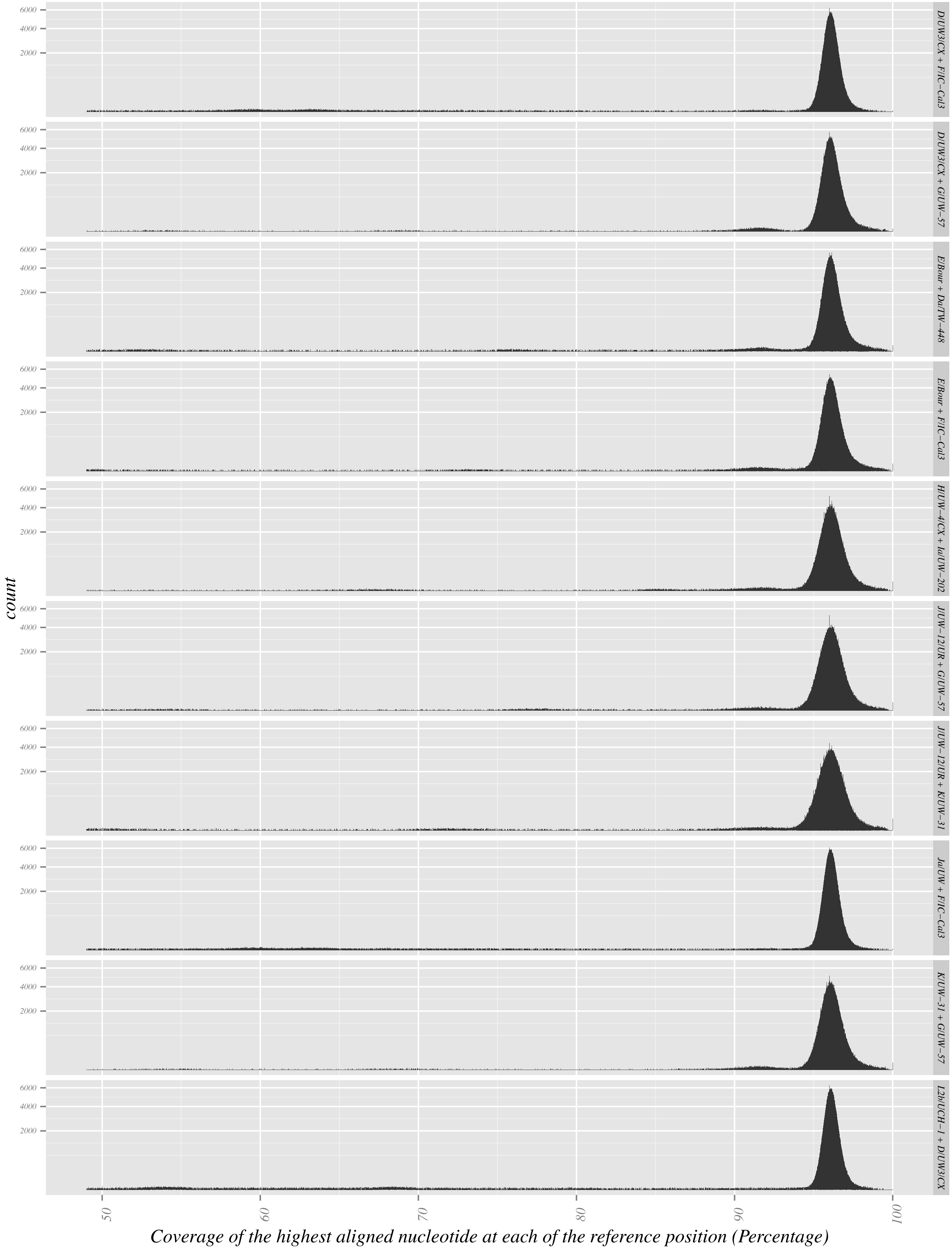

6000
4000
2000
D/UW3/CX + F/IC−Cal3
D/UW3/CX + G/UW−57
E/Bour + Da/TW−448
E/Bour + F/IC−Cal3
H/UW−4/CX + Ia/UW−202
J/UW−12/UR + G/UW−57
J/UW−12/UR + K/UW−31
Ja/UW + F/IC−Cal3
K/UW−31 + G/UW−57
L2b/UCH−1 + D/UW3/CX
count
50
60
70
80
90
100
Coverage of the highest aligned nucleotide at each of the reference position (Percentage)

Figure S4 (b)

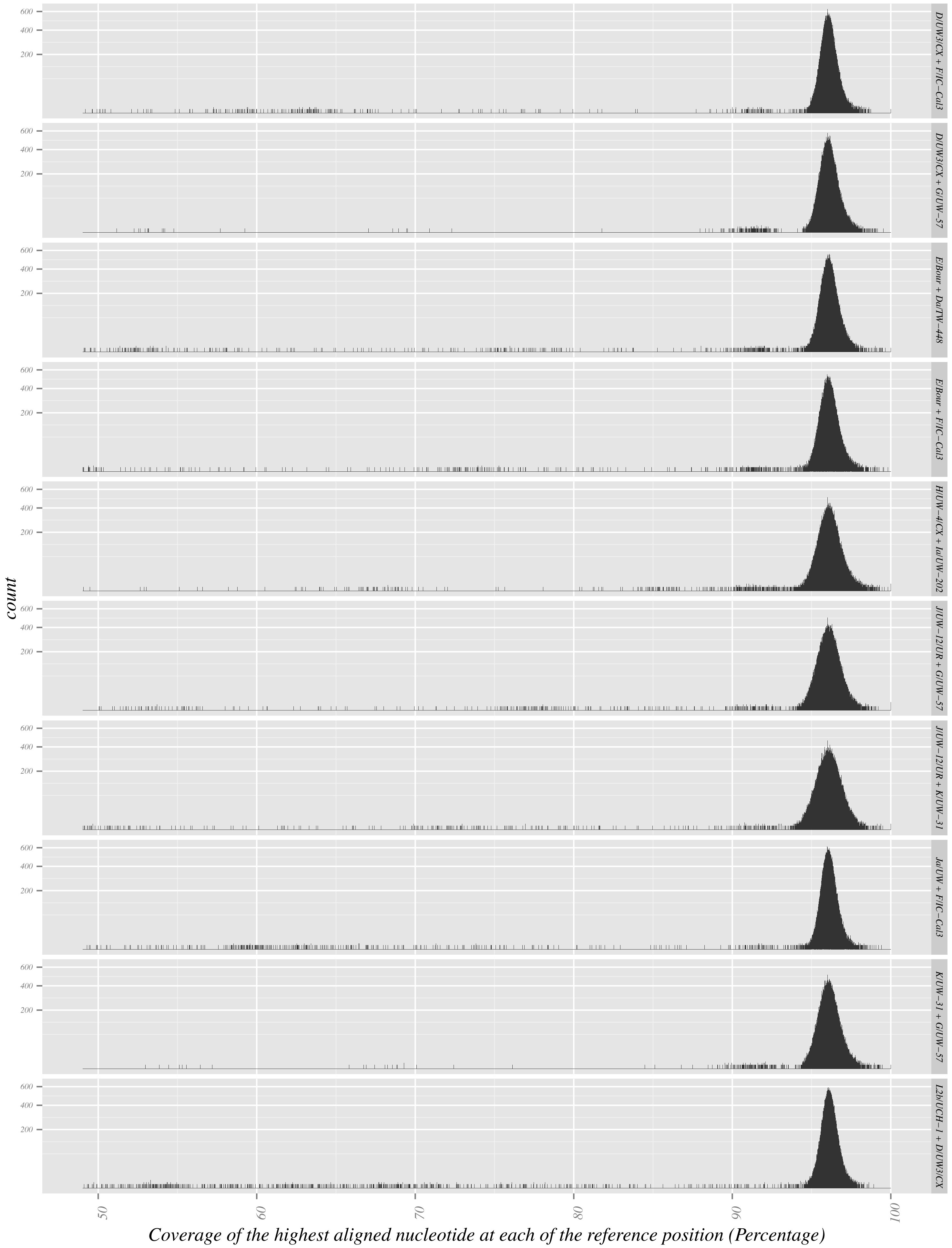

count
D/UW3/CX + F/IC−Cal3
D/UW3/CX + G/UW−57
E/Bour + Da/TW−448
E/Bour + F/IC−Cal3
H/UW−4/CX + Ia/UW−202
J/UW−12/UR + G/UW−57
J/UW−12/UR + K/UW−31
Ja/UW + F/IC−Cal3
K/UW−31 + G/UW−57
L2b/UCH−1 + D/UW3/CX
600
400
200
50
60
70
80
90
100
Coverage of the highest aligned nucleotide at each of the reference position (Percentage)

Figure S5 (a)

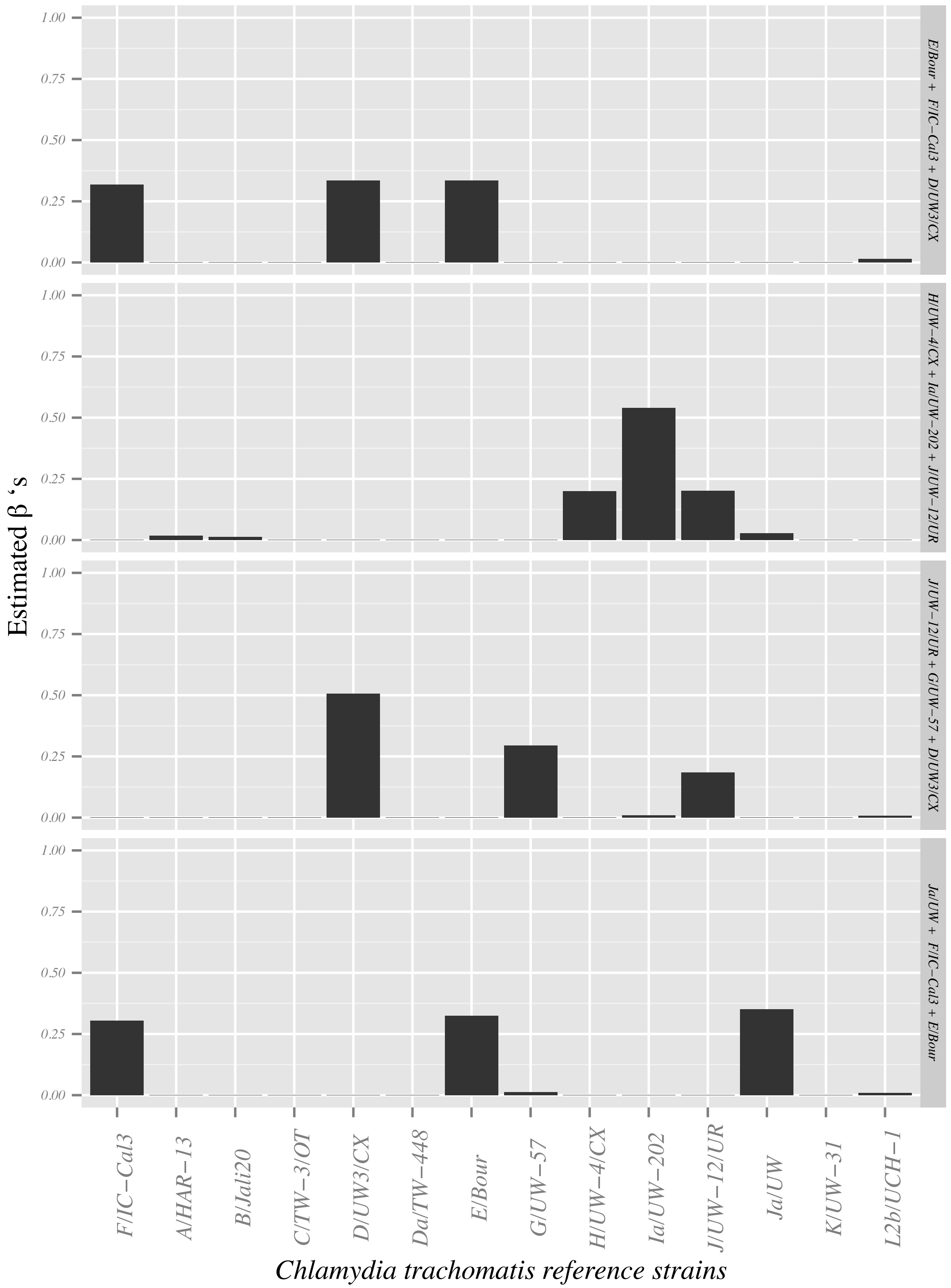
Estimated β 's
1.00
0.75
0.50
0.25
0.00
E/Bour + F/IC−Cal3 + D/UW3/CX
H/UW−4/CX + Ia/UW−202 + J/UW−12/UR
J/UW−12/UR + G/UW−57 + D/UW3/CX
Ja/UW + F/IC−Cal3 + E/Bour
F/IC−Cal3
A/HAR−13
B/Jali20
C/TW−3/OT
D/UW3/CX
Da/TW−448
E/Bour
G/UW−57
H/UW−4/CX
Ia/UW−202
J/UW−12/UR
Ja/UW
K/UW−31
L2b/UCH−1
Chlamydia trachomatis reference strains

Figure S5 (b)

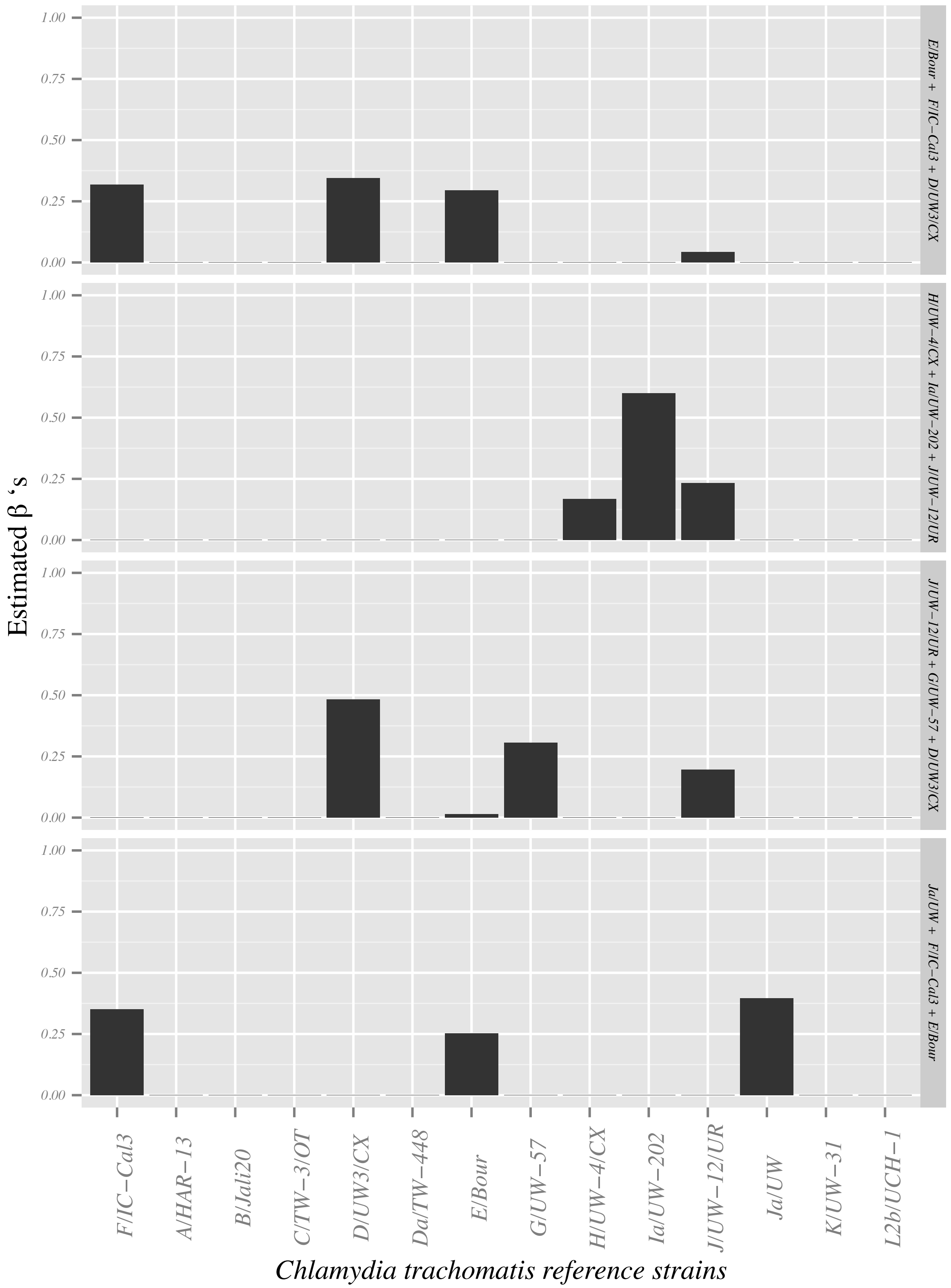
Estimated β 's
1.00
0.75
0.50
0.25
0.00
E/Bour + F/IC−Cal3 + D/UW3/CX
H/UW−4/CX + Ia/UW−202 + J/UW−12/UR
J/UW−12/UR + G/UW−57 + D/UW3/CX
Ja/UW + F/IC−Cal3 + E/Bour
F/IC−Cal3
A/HAR−13
B/Jali20
C/TW−3/OT
D/UW3/CX
Da/TW−448
E/Bour
G/UW−57
H/UW−4/CX
Ia/UW−202
J/UW−12/UR
Ja/UW
K/UW−31
L2b/UCH−1
Chlamydia trachomatis reference strains

Figure S6 (a)

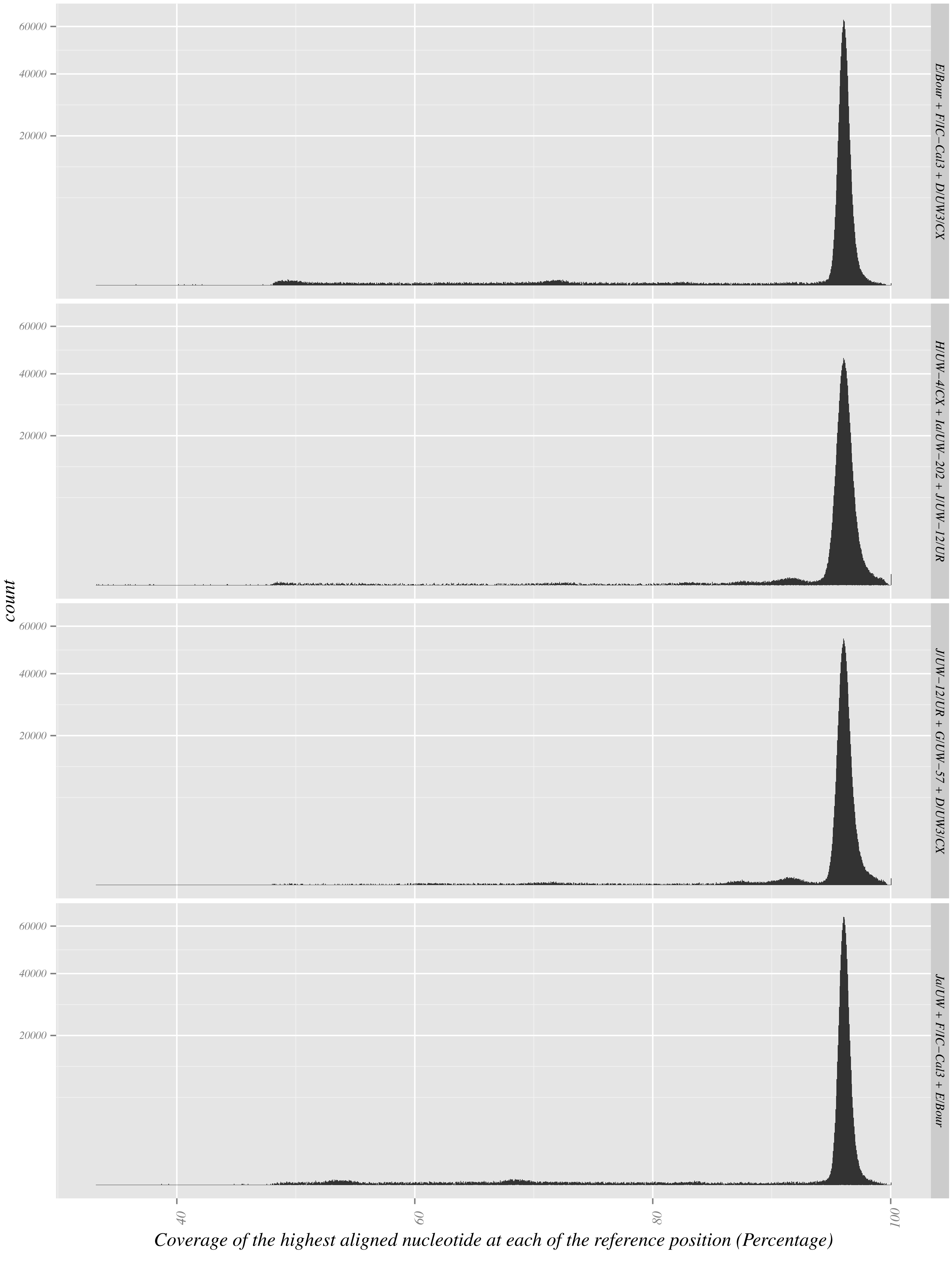

count
60000
40000
20000
E/Bour + F/IC−Cal3 + D/UW3/CX
H/UW−4/CX + Ia/UW−202 + J/UW−12/UR
J/UW−12/UR + G/UW−57 + D/UW3/CX
Ja/UW + F/IC−Cal3 + E/Bour
40
60
80
100
Coverage of the highest aligned nucleotide at each of the reference position (Percentage)

Figure S6 (b)

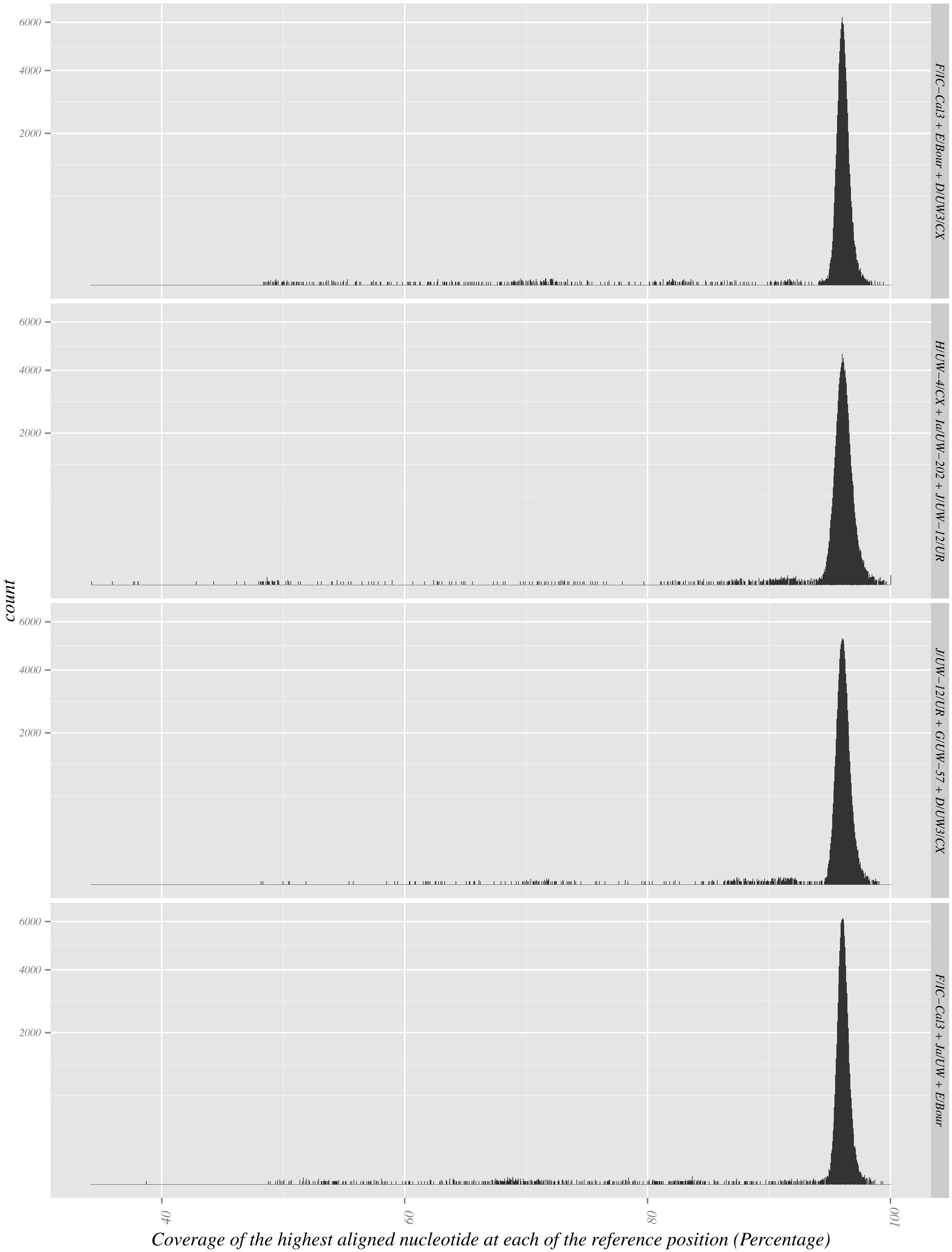

6000
4000
2000
F/IC−Cal3 + E/Bour + D/UW3/CX
H/UW−4/CX + Ia/UW−202 + J/UW−12/UR
J/UW−12/UR + G/UW−57 + D/UW3/CX
F/IC−Cal3 + Ja/UW + E/Bour
count
40
60
80
100
Coverage of the highest aligned nucleotide at each of the reference position (Percentage)

Figure S7.

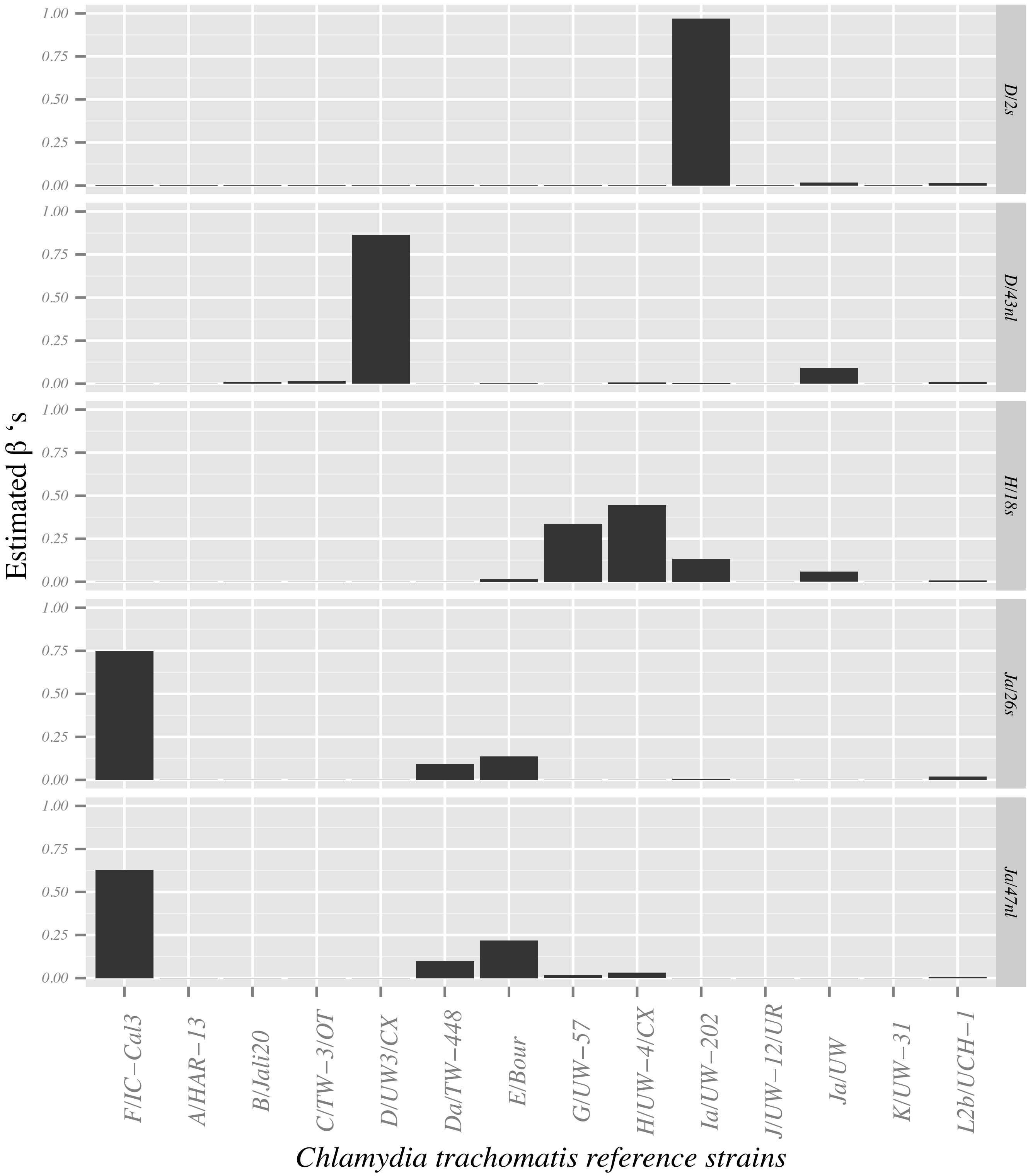

Estimated β 's
1.00
0.75
0.50
0.25
0.00
D/2s
D/43nl
H/18s
Ja/26s
Ja/47nl
F/IC−Cal3
A/HAR−13
B/Jali20
C/TW−3/OT
D/UW3/CX
Da/TW−448
E/Bour
G/UW−57
H/UW−4/CX
Ia/UW−202
J/UW−12/UR
Ja/UW
K/UW−31
L2b/UCH−1
Chlamydia trachomatis reference strains

Figure S8.

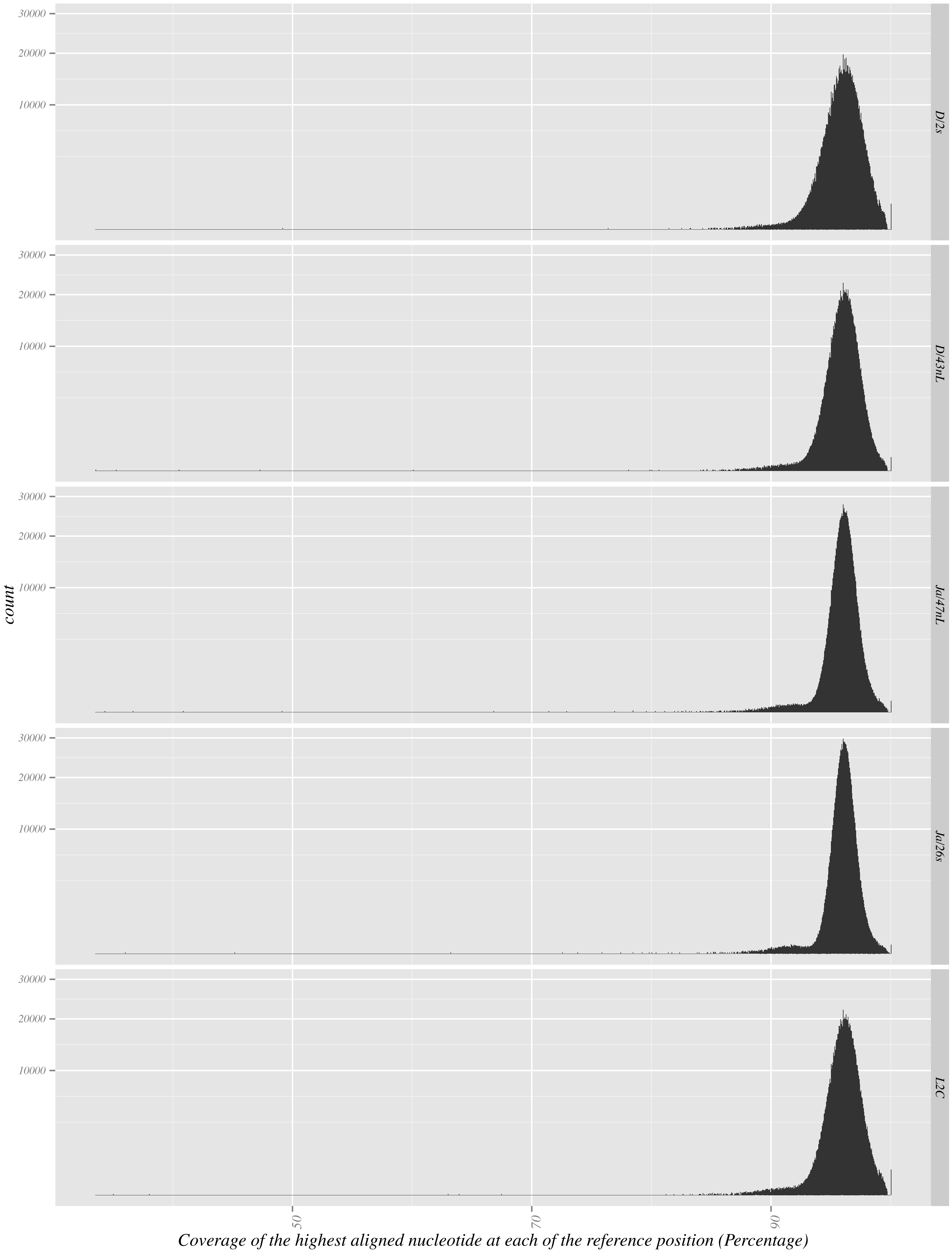
30000
20000
10000
D/2s
D/43nL
Ja/47nL
Ja/26s
L2C
count
50
70
90
Coverage of the highest aligned nucleotide at each of the reference position (Percentage)

Figure S9

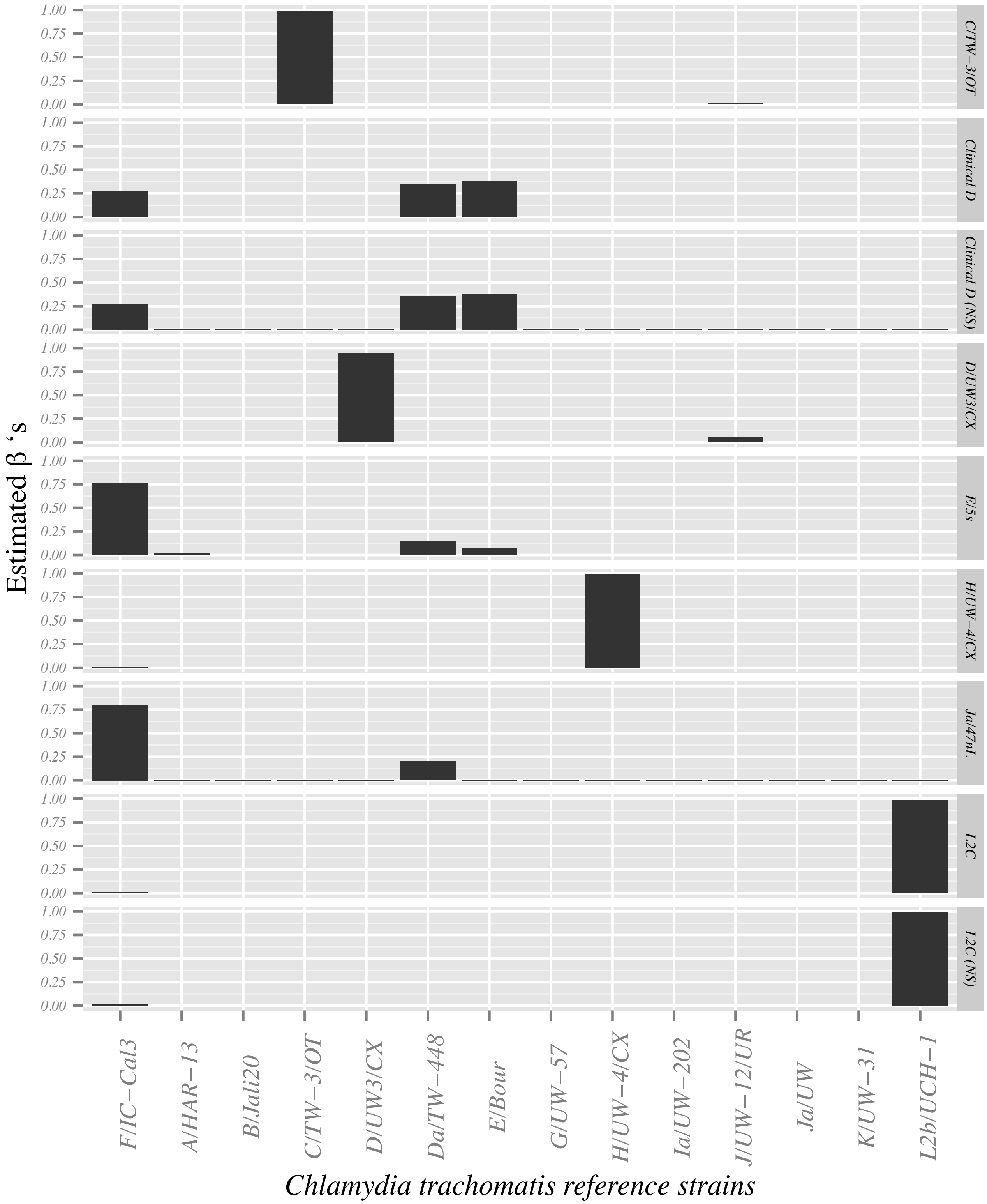

Estimated β ‘s
1.00
0.75
0.50
0.25
0.00
C/TW−3/OT
Clinical D
Clinical D (NS)
D/UW3/CX
E/5s
H/UW−4/CX
Ja/47nL
L2C
L2C (NS)
F/IC−Cal3
A/HAR−13
B/Jali20
C/TW−3/OT
D/UW3/CX
Da/TW−448
E/Bour
G/UW−57
H/UW−4/CX
Ia/UW−202
J/UW−12/UR
Ja/UW
K/UW−31
L2b/UCH−1
Chlamydia trachomatis reference strains

Figure S10

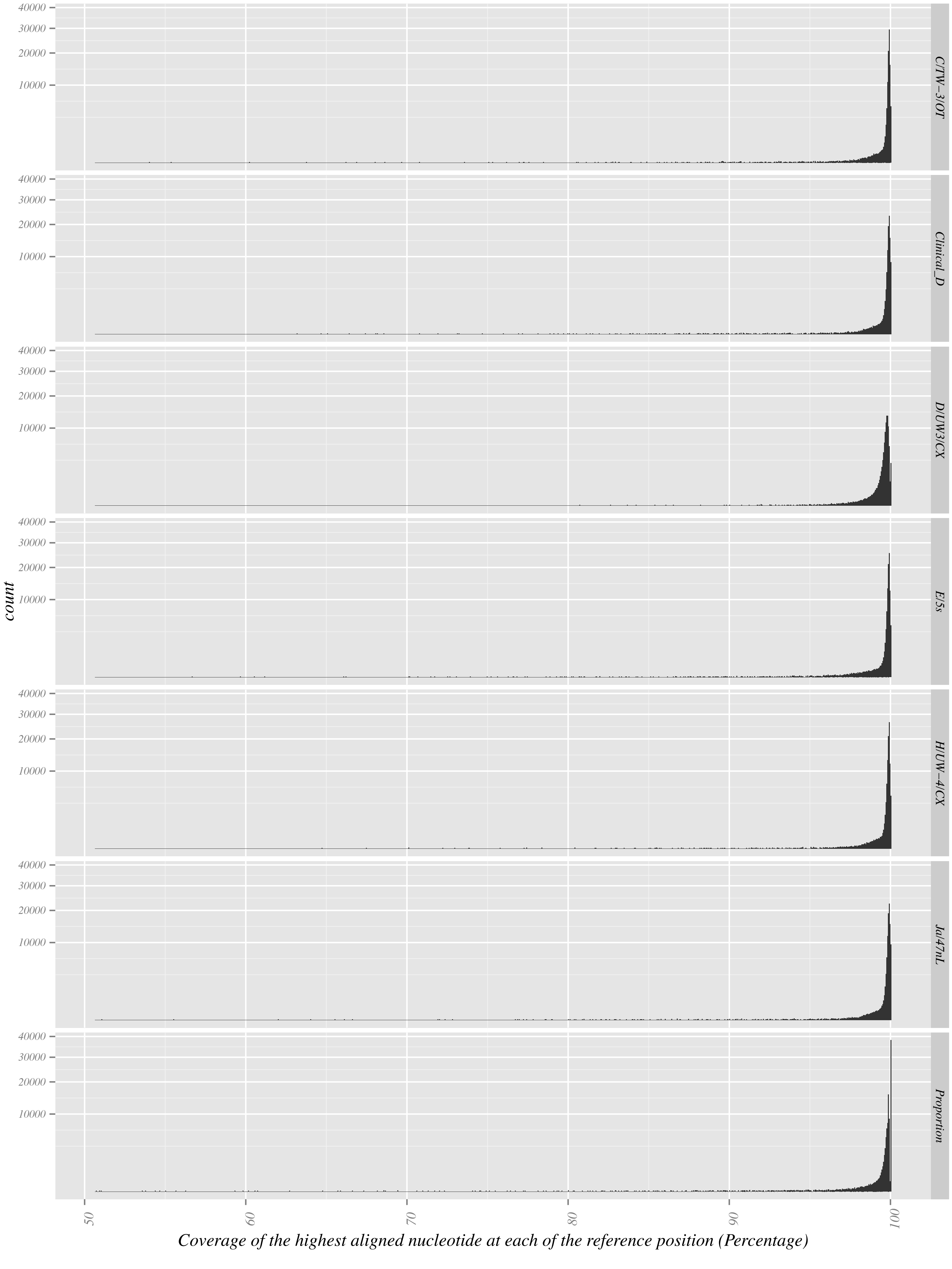
count
40000
30000
20000
10000
C/TW−3/OT
Clinical_D
D/UW3/CX
E/5s
H/UW−4/CX
Ja/47nL
Proportion
50
60
70
80
90
100
Coverage of the highest aligned nucleotide at each of the reference position (Percentage)

Figure S11

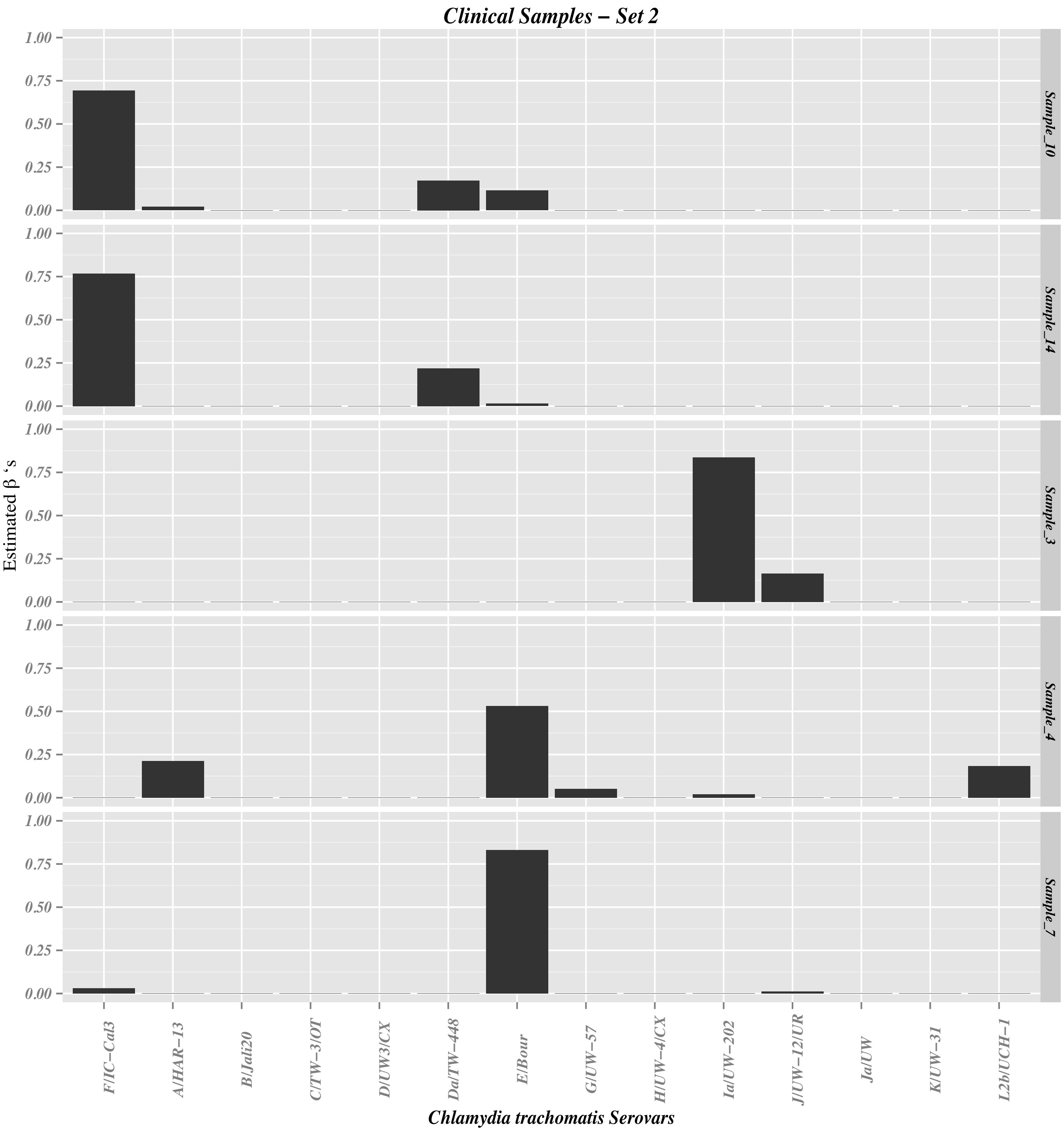
Clinical Samples − Set 2
Estimated β 's
1.00
0.75
0.50
0.25
0.00
Sample_10
Sample_14
Sample_3
Sample_4
Sample_7
F/IC−Cal3
A/HAR−13
B/Jali20
C/TW−3/OT
D/UW3/CX
Da/TW−448
E/Bour
G/UW−57
H/UW−4/CX
Ia/UW−202
J/UW−12/UR
Ja/UW
K/UW−31
L2b/UCH−1
Chlamydia trachomatis Serovars

Figure S12

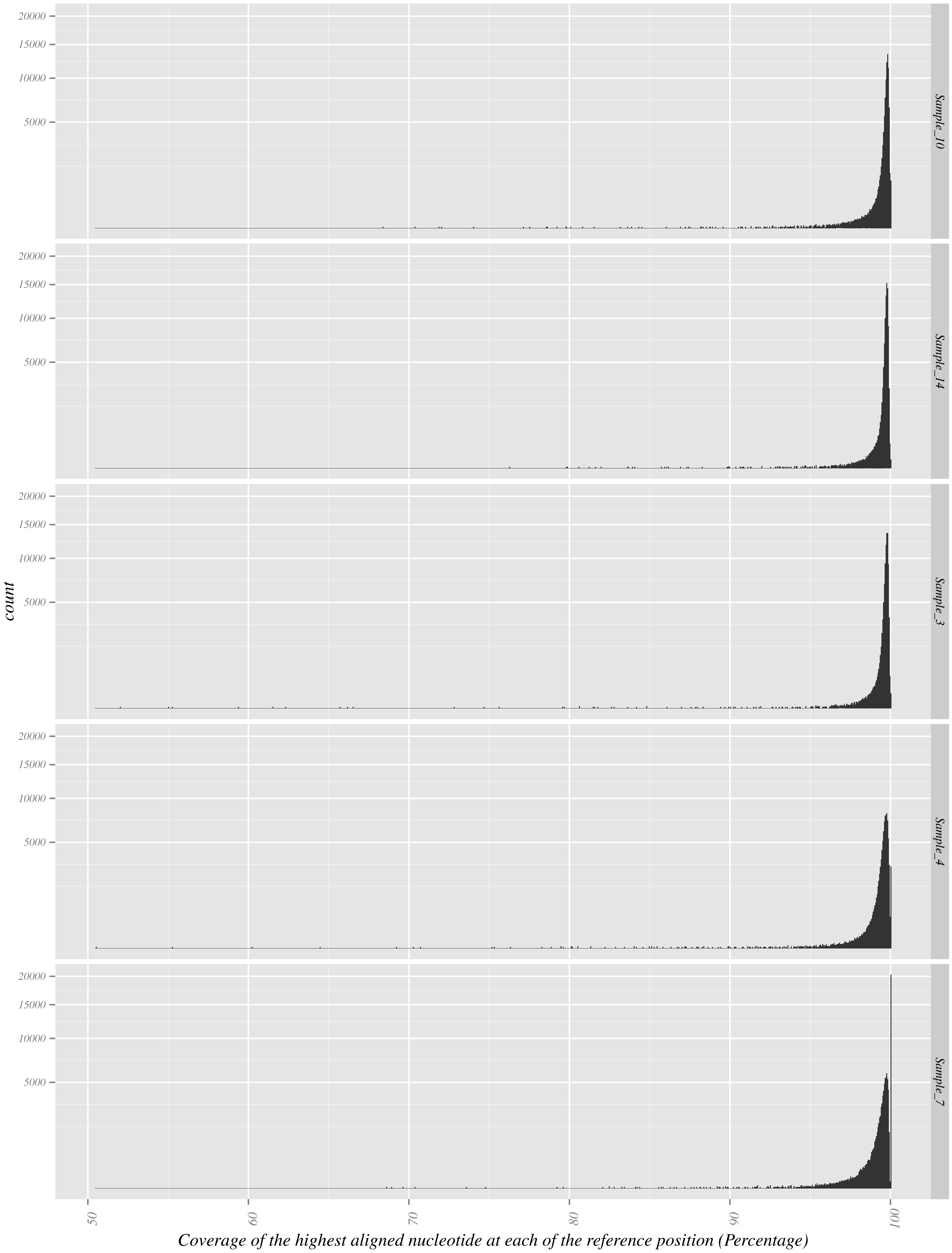

20000
15000
10000
5000
Sample_10
Sample_14
Sample_3
Sample_4
Sample_7
count
50
60
70
80
90
100
Coverage of the highest aligned nucleotide at each of the reference position (Percentage)

Figure S13

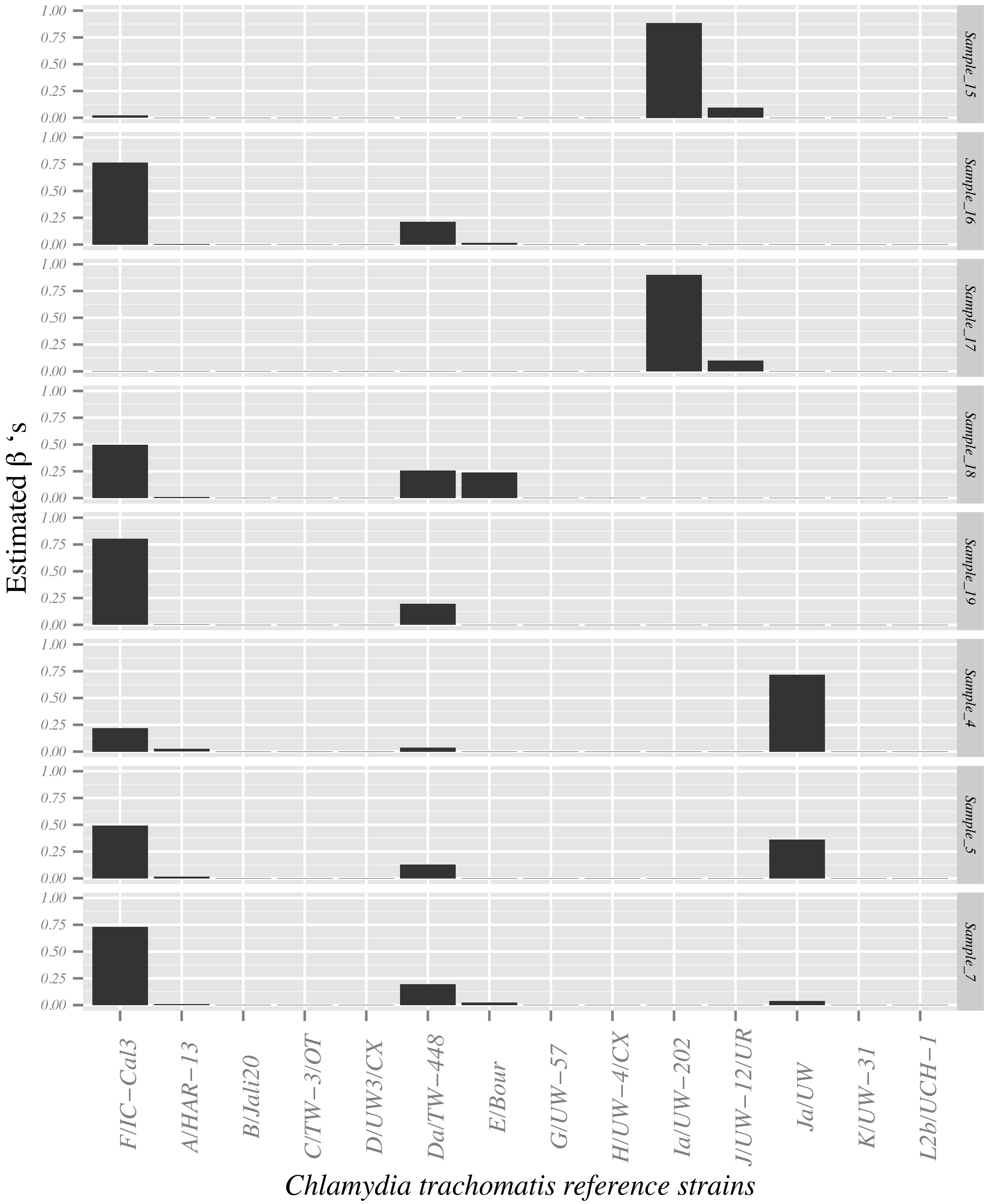

Figure S14

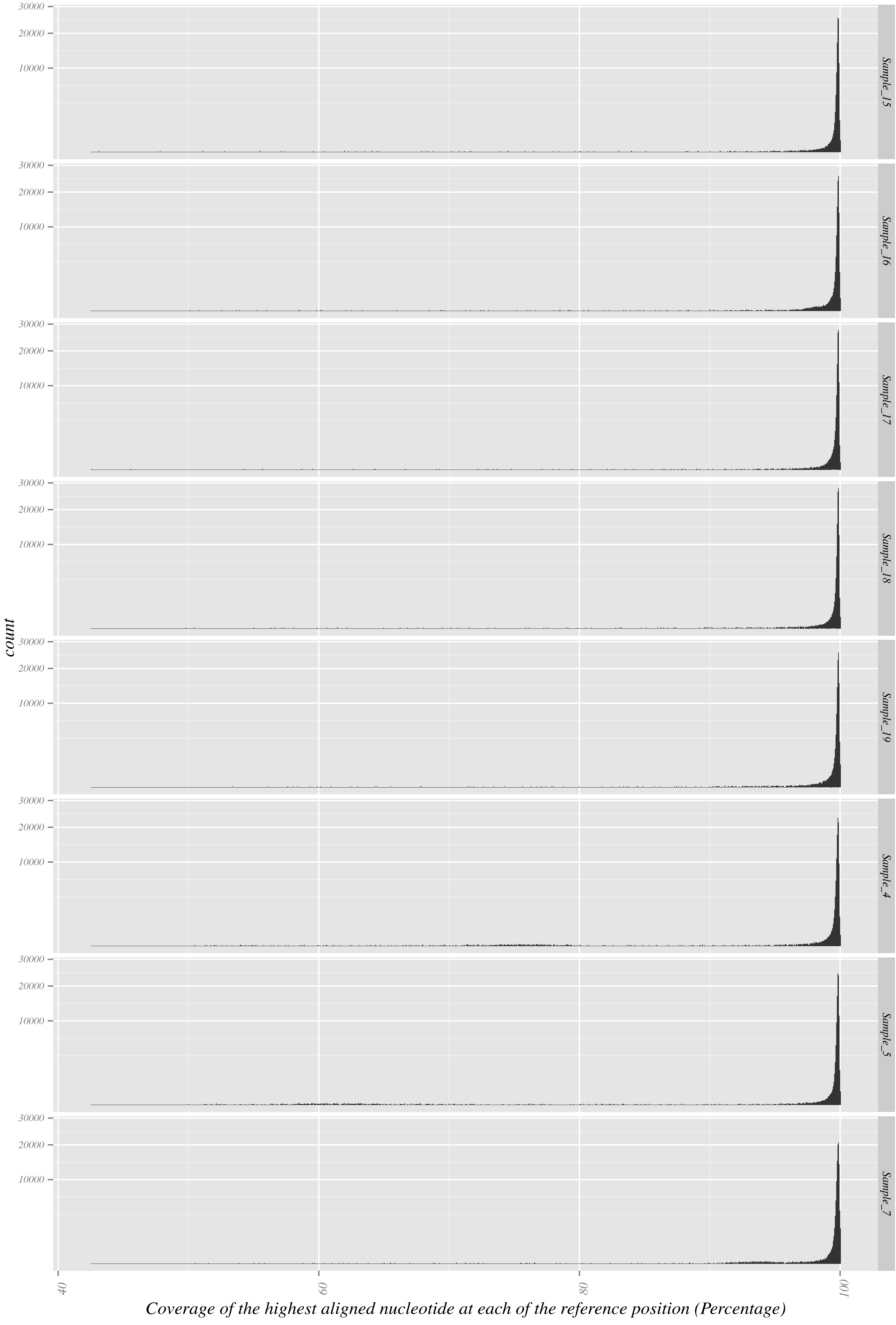

30000
20000
10000
count
Sample_15
Sample_16
Sample_17
Sample_18
Sample_19
Sample_4
Sample_5
Sample_7
40
60
80
100
Coverage of the highest aligned nucleotide at each of the reference position (Percentage)

Figure S15

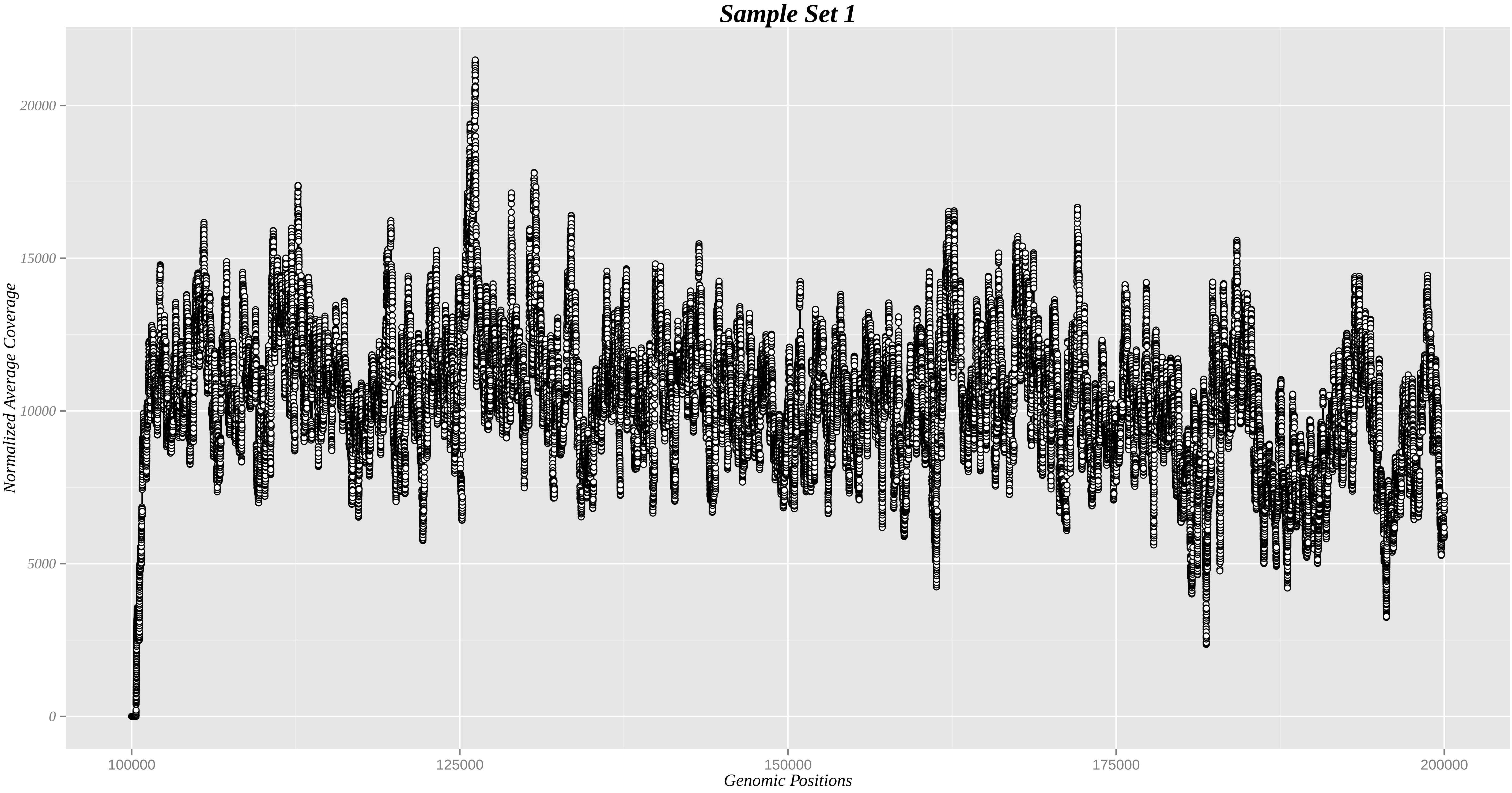
Sample Set 1
Normalized Average Coverage
20000
15000
10000
5000
0
100000
125000
150000
175000
200000
Genomic Positions

Figure S16

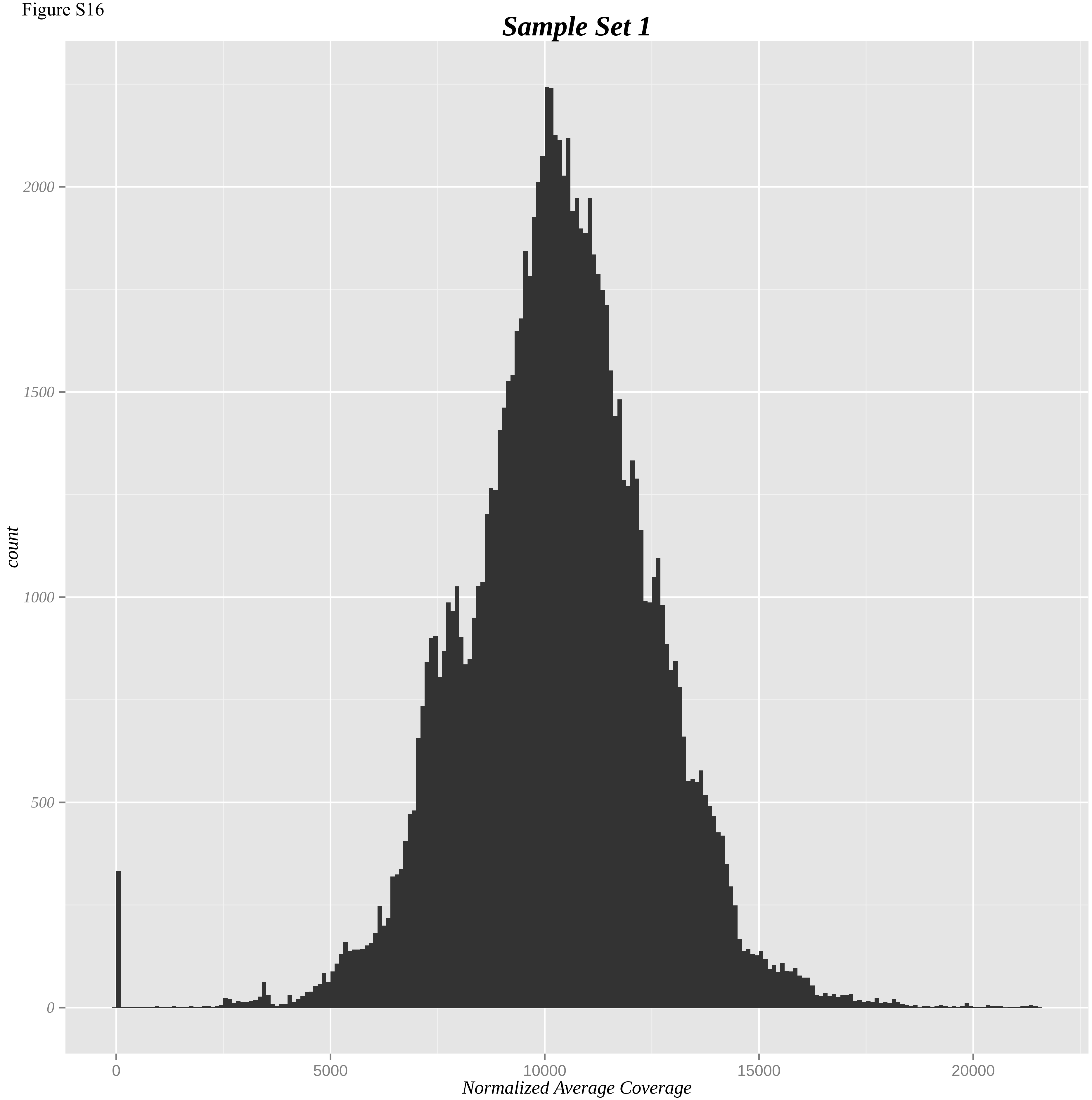
Sample Set 1
count
2000
1500
1000
500
0
0
5000
10000
15000
20000
Normalized Average Coverage

Figure S17

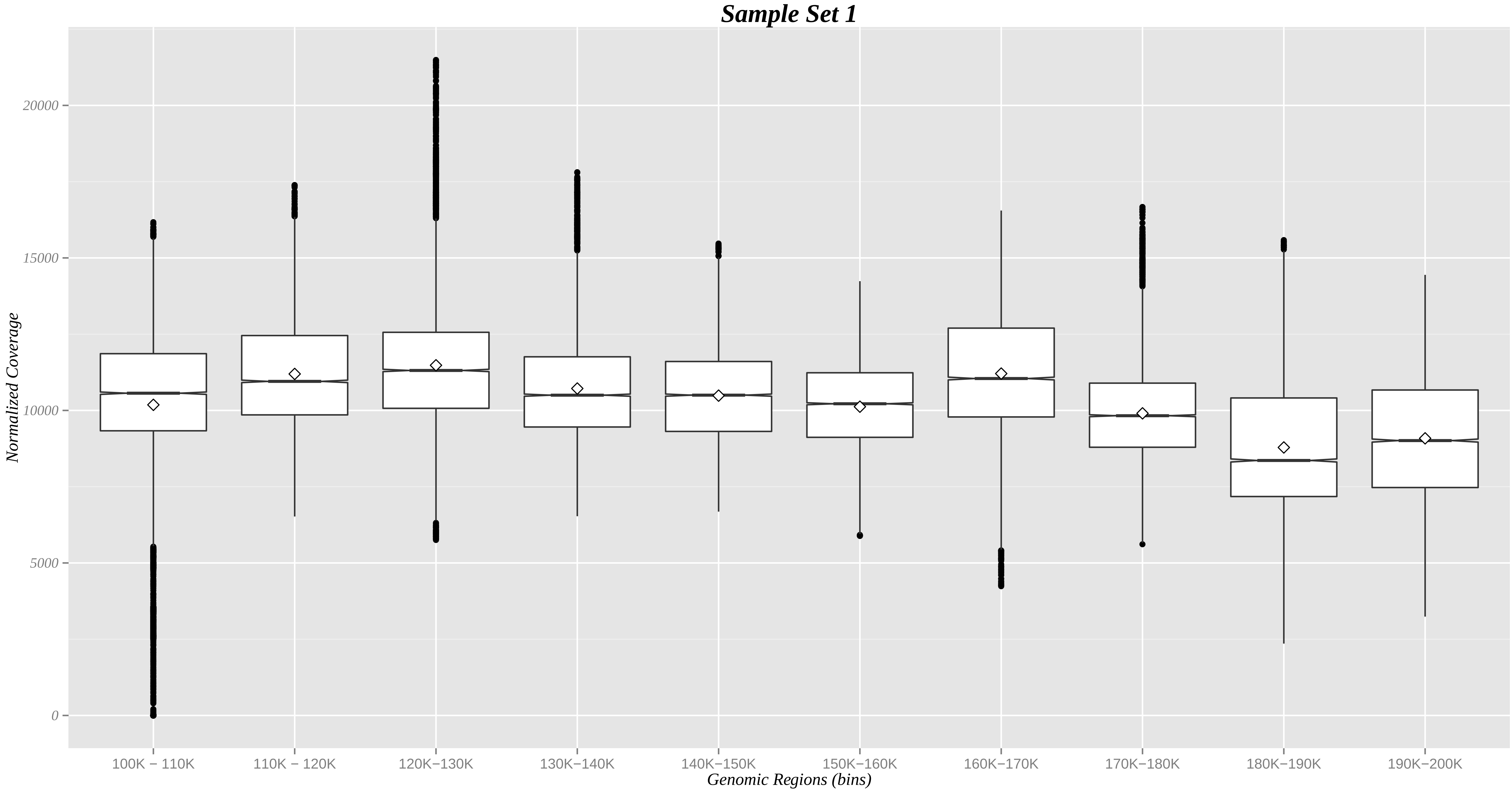
Sample Set 1
Normalized Coverage
20000
15000
10000
5000
0
100K − 110K
110K − 120K
120K−130K
130K−140K
140K−150K
150K−160K
160K−170K
170K−180K
180K−190K
190K−200K
Genomic Regions (bins)

Figure S18

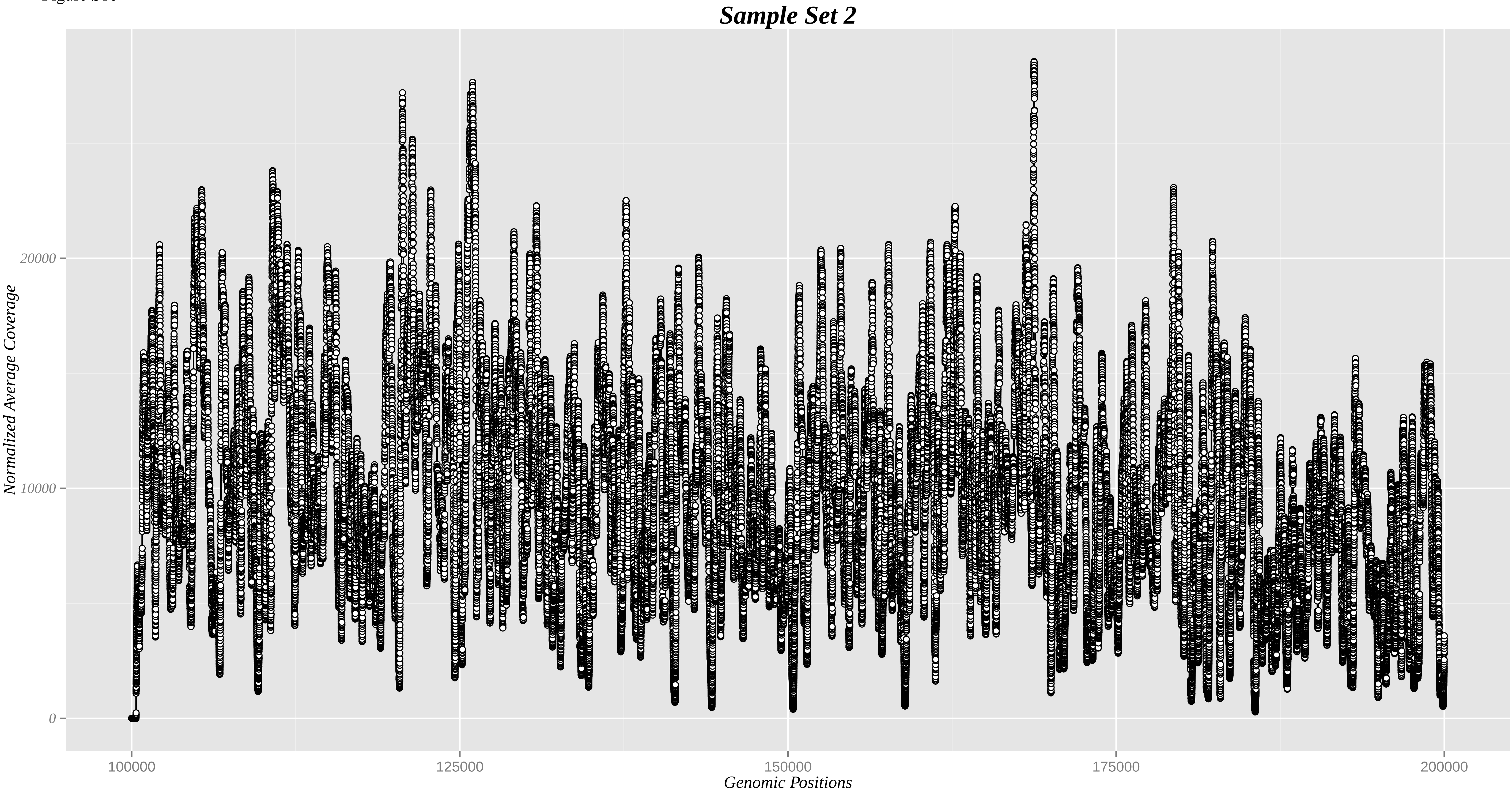
Sample Set 2
Normalized Average Coverage
20000
10000
0
100000
125000
150000
175000
200000
Genomic Positions

Figure S19

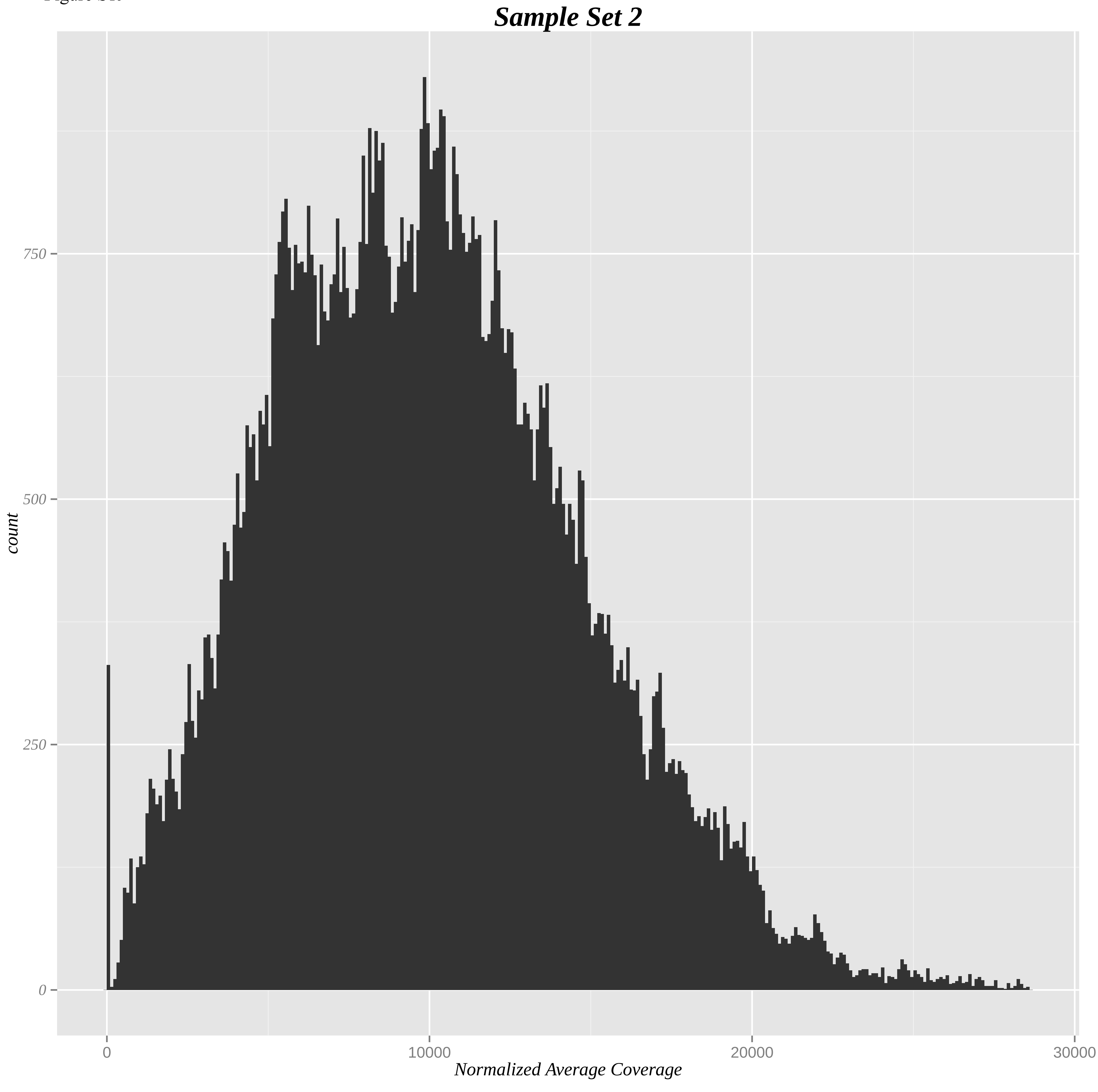
Sample Set 2
count
750
500
250
0
0
10000
20000
30000
Normalized Average Coverage

Figure S20

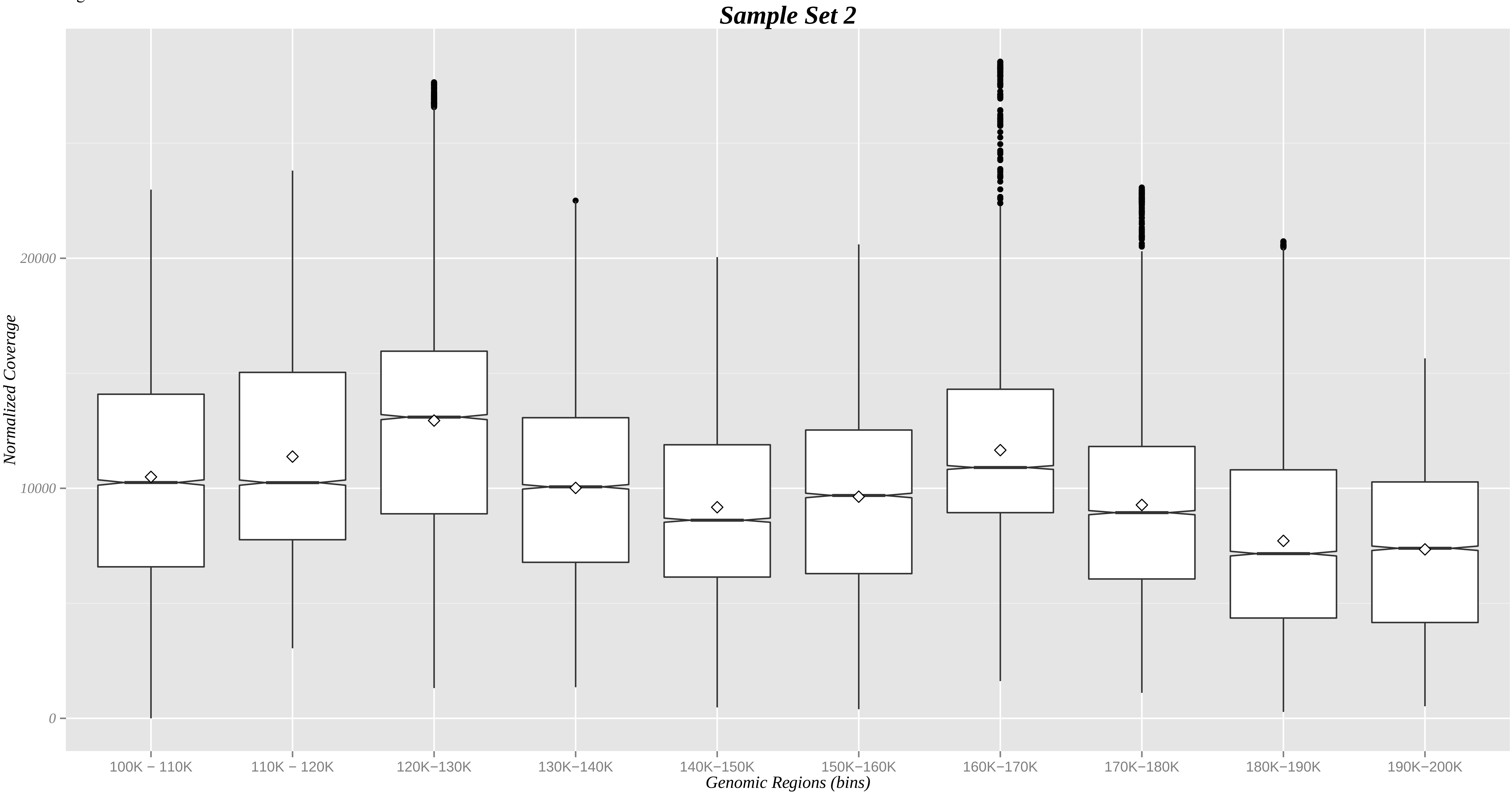

Sample Set 2
Normalized Coverage
20000
10000
0
100K − 110K
110K − 120K
120K−130K
130K−140K
140K−150K
150K−160K
160K−170K
170K−180K
180K−190K
190K−200K
Genomic Regions (bins)

Figure S21

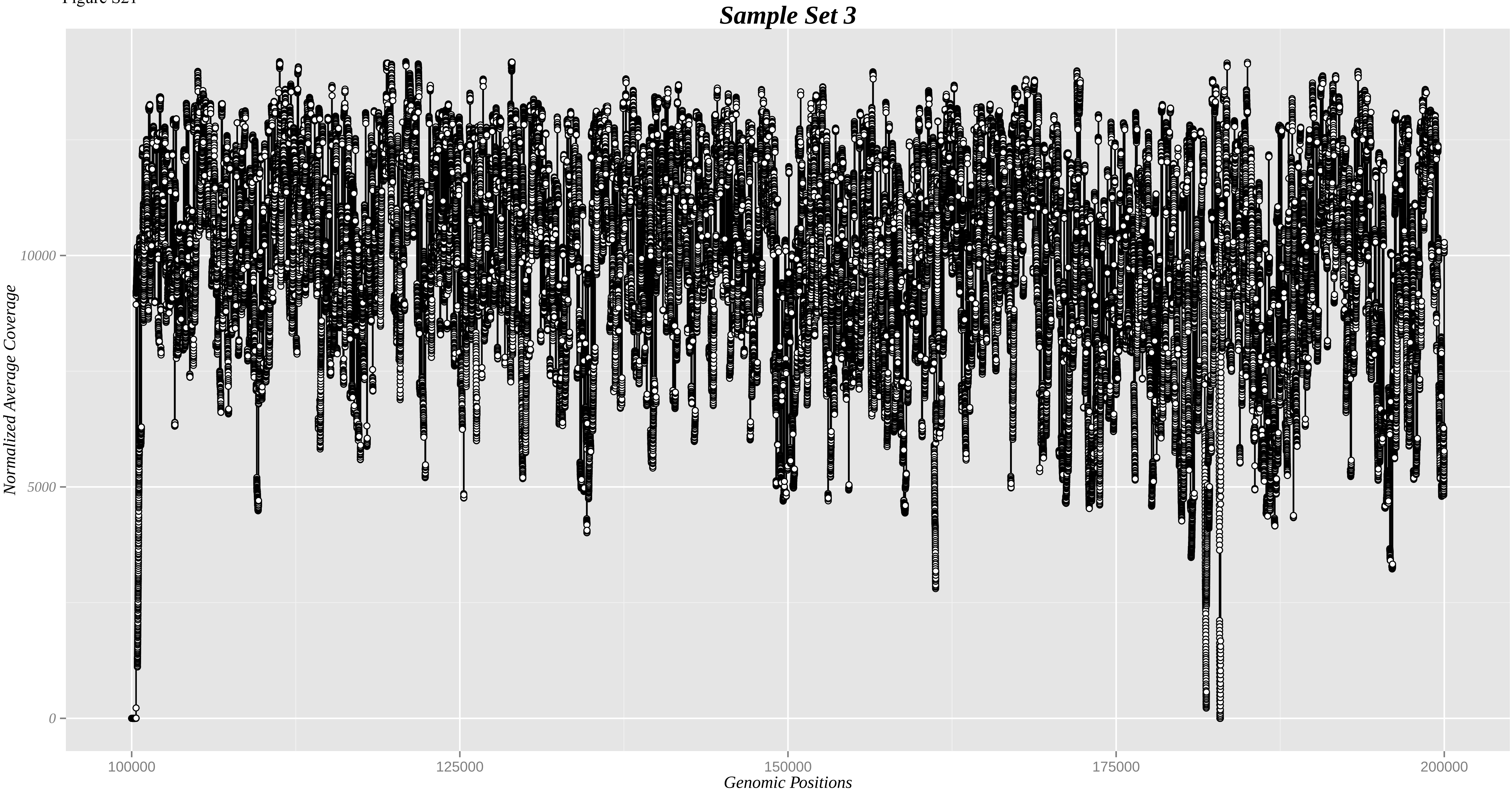
Sample Set 3
Normalized Average Coverage
0
5000
10000
100000
125000
150000
175000
200000
Genomic Positions

Figure S22

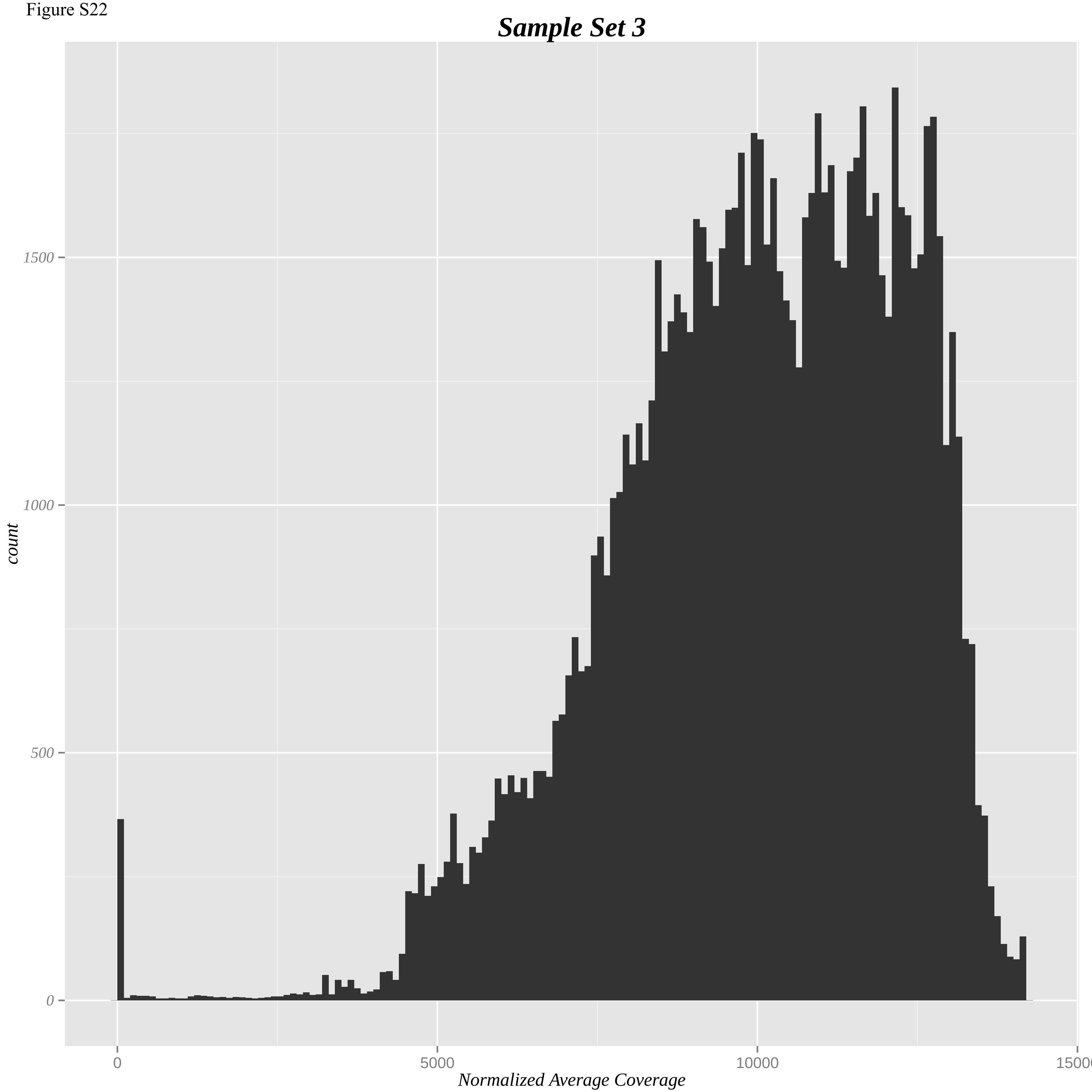

Sample Set 3
count
1500
1000
500
0
0
5000
10000
15000
Normalized Average Coverage

Figure S23

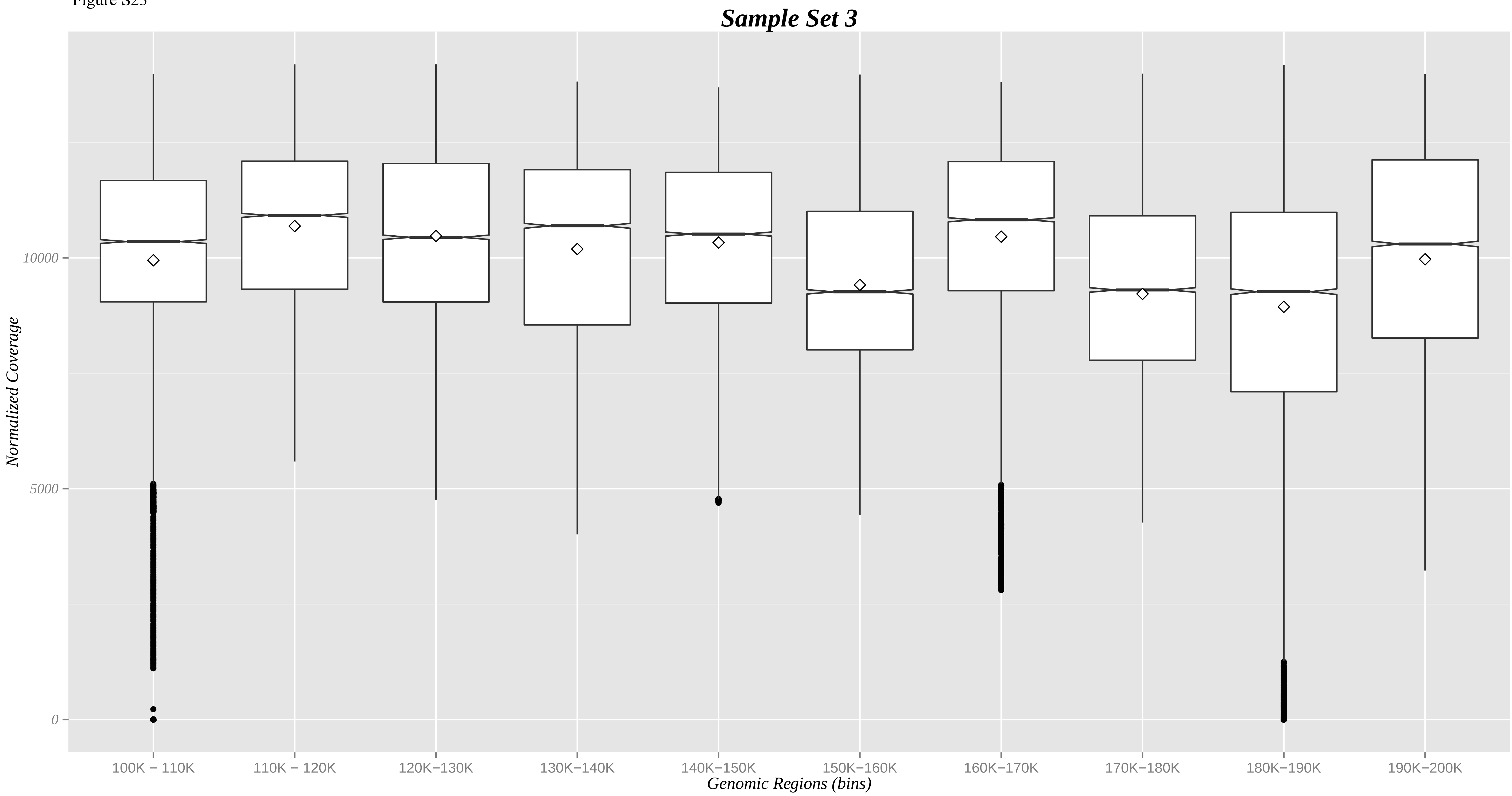
Sample Set 3
Normalized Coverage
10000
5000
0
100K − 110K
110K − 120K
120K−130K
130K−140K
140K−150K
150K−160K
160K−170K
170K−180K
180K−190K
190K−200K
Genomic Regions (bins)

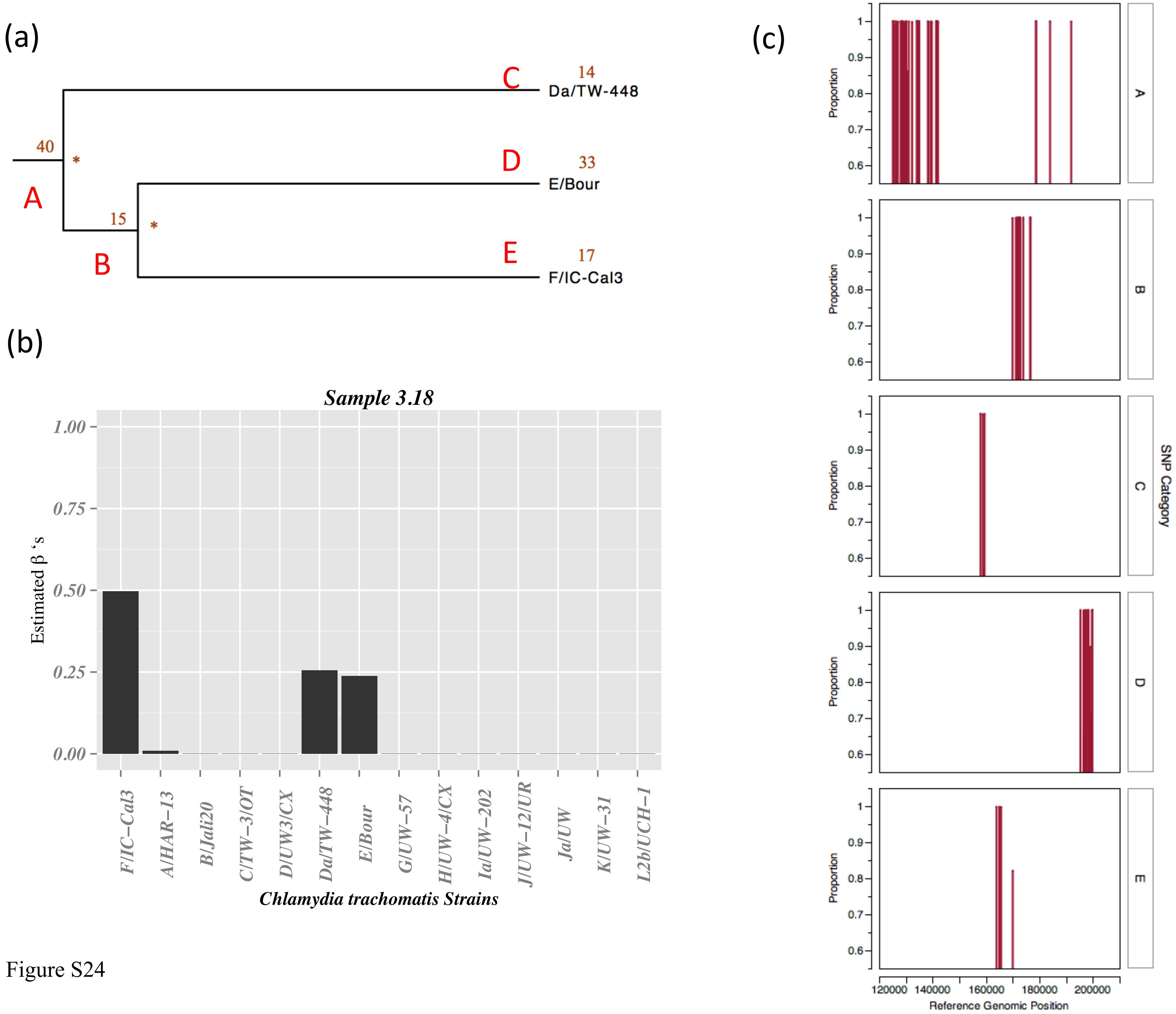
(a)
C
14
Da/TW-448
40
*
D
33
E/Bour
A
15
*
B
E
17
F/IC-Cal3
(b)
Sample 3.18
Estimated β ´s
1.00
0.75
0.50
0.25
0.00
F/IC–Cal3
A/HAR–13
B/Jali20
C/TW–3/OT
D/UW3/CX
Da/TW–448
E/Bour
G/UW–57
H/UW–4/CX
Ia/UW–202
J/UW–12/UR
Ja/UW
K/UW–31
L2b/UCH–1
Chlamydia trachomatis Strains
(c)
Proportion
SNP Category
A
B
C
D
E
120000
140000
160000
180000
200000
Reference Genomic Position

Figure S24

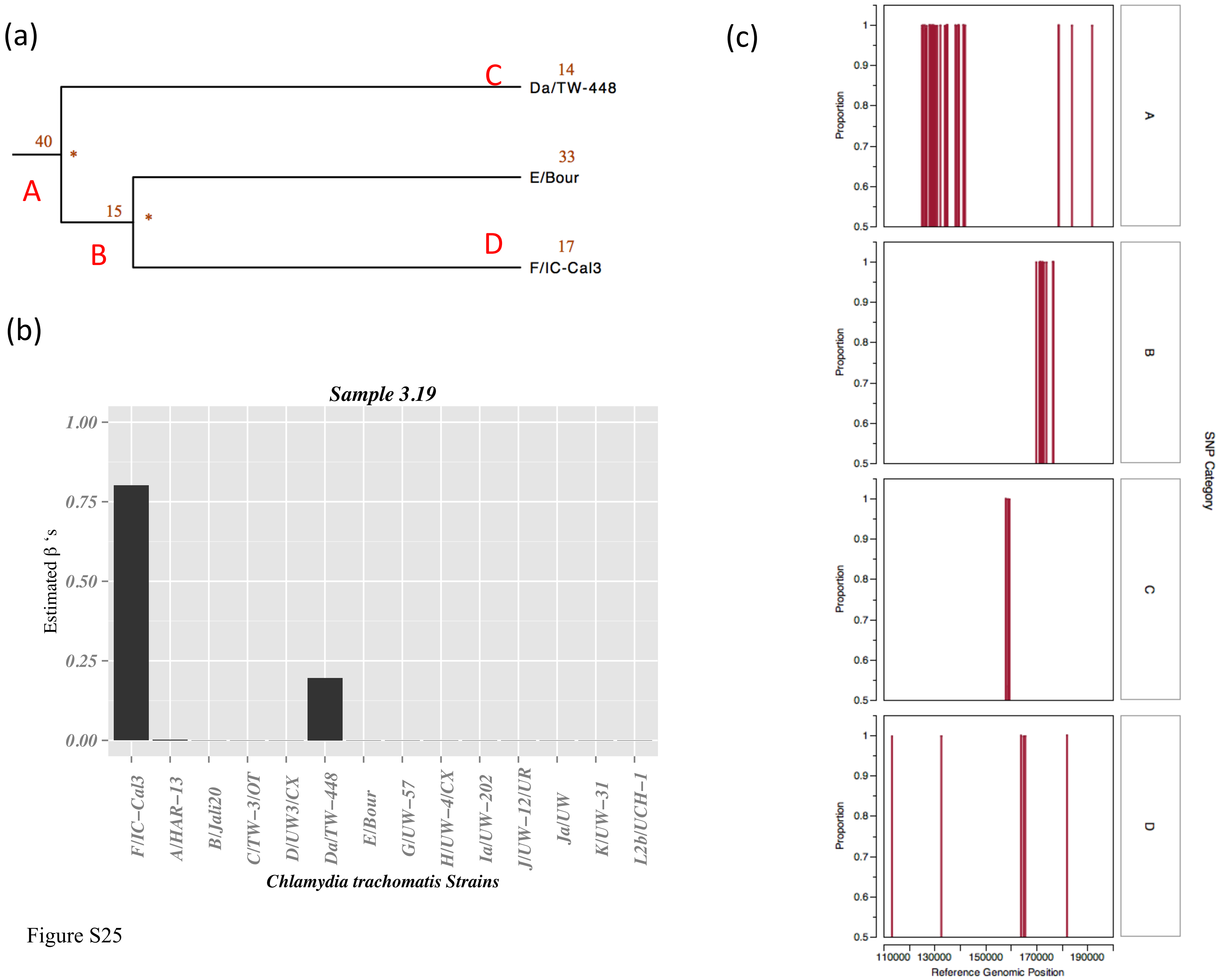
(a)
40
A
15
B
C
14
Da/TW-448
33
E/Bour
D
17
F/IC-Cal3
(b)
Sample 3.19
Estimated β 's
1.00
0.75
0.50
0.25
0.00
F/IC−Cal3
A/HAR−13
B/Jali20
C/TW−3/OT
D/UW3/CX
Da/TW−448
E/Bour
G/UW−57
H/UW−4/CX
Ia/UW−202
J/UW−12/UR
Ja/UW
K/UW−31
L2b/UCH−1
Chlamydia trachomatis Strains
(c)
Proportion
A
B
C
D
SNP Category
1
0.9
0.8
0.7
0.6
0.5
110000
130000
150000
170000
190000
Reference Genomic Position

Figure S25

Supplementary Table S1. List of *C. trachomatis* genomes used for primer design, ancestral sequence regeneration and whole genome MAUVE alignment to generate the SNP pattern file used in this study.

Strain name	Serotype	Strains used in primer design	Strains used in ancestral sequence regeneration	Strains used for generating the SNP pattern file for *binstrain* analysis	Accession
A/HAR-13*	A	X	X	X	CP000051
B/TZ1A828/OT	B	X	X	-	FM872308
B/Jali20	B	X	-	X	FM872307
C/TW-3/OT*	C	-	-	X	SRA051538.1
D/UW3/CX*	D	X	X	X	AE001273
E/11023	E	X	X	-	CP001890
Fs(70)	F	X	X	-	ABYF01000001
F/IC-Cal3*	F	-	-	X	Not Published
G/9301	G	X	X	-	CP001930
G/9768	G	X	-	-	CP001887
G/11222	G	X	-	-	CP001888
G/11074	G	X	-	-	CP001889
G/UW-57*	G	-	-	X	SRA051545.1
H/UW-4/CX*	H	-	-	X	SRA051548.1
Ia/UW-202*	Ia	-	-	X	SRA051537.1
L2/434/BU	L2	X	X	-	AM884176
L2b/UCH-1	L2b	X	X	X	ERR008581
Da/TW-448*	Da	-	-	X	Not Published
E/Bour*	E	-	-	X	NC_020971
J/UW-12/UR*	J	-	-	X	Not Published
Ja/UW*	Ja	-	-	X	Not Published
K/UW-31*	K	-	-	X	Not Published

* indicates reference *Chlamydia trachomatis* strains

Supplementry Table S2. Results from the simulated *C. trachomatis* uni, bi and tri artifical mixed infected samples				
**Uni sample (single strain Samples)**				
**Sample Name**	***C. trachomatis* Reference strain simulated and/or merged (Simulated Coverage)**	**Percentage/proportions of the simulated strain present in each sample**	**Binomial β estimates for whole genome simulation (Strain/s with the highest β estimate/s)**	**Binomial β estimates for the100kb targeted simulation (Strain/s with the highest β estimate/s)**
Uni sample 1	D/UW-3/CX (5000X)	100%	0.9785 (D/UW-3/CX)	0.98056 (D/UW-3/CX)
Uni sample 2	E/Bour (3000X)	100%	0.9618 (E/Bour)	0.9621(E/Bour)
Uni sample 3	Da/TW-448 (6500X)	100%	0.9591 (Da/TW-448)	0.9969 (Da/TW-448)
Uni sample 4	F/IC-Cal3 (5500X)	100%	0.9466 (F/IC-Cal3)	0.9742 (F/IC-Cal3)
Uni sample 5	Ja/UW (6000X)	100%	0.9546 (Ja/UW)	0.9839 (Ja/UW)
**Bi Mixture Samples (Two strain Samples)**				
Bi Mixture 1	L2b/UCH-1 (4000X) + D/UW-3/CX (5000X)	44.4% + 55.55%	0.4488 (D/UW-3/CX); 0.5262 (L2b/UCH-1)	0.5242 (D/UW-3/CX); 0.4702 (L2b/UCH-1)
Bi Mixture 2	J/UW-12/UR (1500X) + K/UW-31 (2500X)	37.5% + 62.5%	0.3394 (J/UW-12/UR); 0.5981 (K/UW-31)	0.3628 (J/UW-12/UR); 0.6314 (K/UW-31)
Bi Mixture 3	D/UW-3/CX (5000X) +G/UW-57 (3500X)	58.8% + 41.2%	0.607 (D/UW-3/CX); 0.3690 (G/UW-57)	0.5703 (D/UW-3/CX); 0.3989 (G/UW-57)
Bi Mixture 4	H/UW-4/CX (1000X) + Ia/UW-202 (4500X)	18.18% + 81.81%	0.2339 (H/UW-4/CX); 0.7114 (Ia/UW-202)	0.1892 (H/UW-4/CX); 0.7250 (Ia/UW-202)
Bi Mixture 5	D/UW-3/CX (5000X) + F/IC-Cal3 (5500X)	47.62% + 52.38%	0.4579 (D/UW-3/CX); 0.5012 (F/IC-Cal3)	0.4972 (D/UW-3/CX); 0.4804 (F/IC-Cal3)
Bi Mixture 6	E/Bour (3000X) + F/IC-Cal3 (5500X)	35.30% + 64.70%	0.5016 (E/Bour); 0.4817 (F/IC-Cal3)	0.5884 (E/Bour); 0.4115 (F/IC-Cal3)
Bi Mixture 7	Ja/UW (6000X) + F/IC-Cal3 (5500X)	52.17% + 47.82%	0.4695 (Ja/UW); 0.5110 (F/IC-Cal3)	0.5207 (Ja/UW); 0.4792 (F/IC-Cal3)
Bi Mixture 8	E/Bour (3000X) + Da/TW-448 (6500X)	31.57% + 68.42%	0.4625 (E/Bour); 0.5050 (Da/TW-448)	0.3285 (E/Bour); 0.6687 (Da/TW-448)
Bi Mixture 9	K/UW-31 (2500X) + G/UW-57 (3500X)	41.66% + 58.33%	0.3621 (K/UW-31); 0.6197 (G/UW-57)	0.4177(K/UW-31); 0.5579 (G/UW-57)
Bi Mixture 10	J/UW-12/UR (1500X) + G/UW-57 (3500X)	30% + 70%	0.2327 (J/UW-12/UR); 0.6690 (G/UW-57)	0.3048 (J/UW-12/UR); 0.6823 (G/UW-57)
**Tri Mixture Samples**				
Tri Mixtue 1	Ja/UW (6000X) + F/IC-Cal3 (5500X) + E/Bour (3000X)	41.37% + 37.93% + 20.68%	0.3508 (Ja/UW); 0.3044 (F/IC-Cal3); 0.3239 (E/Bour)	0.3958 (Ja/UW); 0.3508 (F/IC-Cal3); 0.2533 (E/Bour)
Tri Mixtue 2	F/IC-Cal3 (5500X) + E/Bour (3000X) + D/UW-3/CX (5000X)	40.7% + 22.22% + 37.03%	0.3172 (F/IC-Cal3); 0.3336 (E/Bour); 0.3343 (D/UW-3/CX)	0.3181 (F/IC-Cal3); 0.2942 (E/Bour); 0.3443 (D/UW-3/CX)
Tri Mixtue 3	J/UW-12/UR (1500X) + G/UW-57 (3500X) + D/UW-3/CX (5000X)	15% + 35% + 50%	0.1839 (J/UW-12/UR); 0.2945 (G/UW-57); 0.5053 (D/UW-3/CX)	0.1952 (J/UW-12/UR); 0.3068 (G/UW-57); 0.4829 (D/UW-3/CX)
Tri Mixtue 4	H/UW-4/CX (1000X) + Ia/UW-202 (4500X) + J/UW-12/UR (1500X)	14.28% + 64.28% + 21.42%	0.1985 (H/UW-4/CX); 0.5395 (Ia/UW-202); 0.2011 (J/UW-12/UR	0.1673 (H/UW-4/CX); 0.5992 (Ia/UW-202); 0.2333 (J/UW-12/UR)
**Recombinant Samples**	***C. trachomatis* recombinant strains simulated (Simulated Coverage) (Strains involved in recombination identified via MLST)**			
Recombinant Strain 1	D/2s (1000X) 1(D/UW3/CX & Ia/UW-202)	100%	0.9688 (Ia/UW-202); 0.0137 (L2b/UCH-1); 0.0179 (Ja/UW)	NA
Recombinant Strain 2	D/43nL (1500X) (D/UW3/CX & G/UW-57)	100%	0.8631 (D/UW3/CX)	NA
Recombinant Strain 3	H/18s (2000X) (H/UW-4/CX & G/UW-57)	100%	0.446807286 (H/UW-4/CX); 0.335769141 (G/UW-57)	NA
Recombinant Strain 4	Ja/26s (3000X) (Ja/UW, Da/TW-448 &E/Bour)	100%	0.135279275 (E/Bour); 0.749444316 (F/IC-Cal3)	NA
Recombinant Strain 5	Ja/47nL (2500X) (Ja/UW & F/IC-Cal3)	100%	0.21836503 (E/Bour); 0.62930026 (F/IC-Cal3); 0.099313389 (Da/TW-448)	NA
Recombinant Strain 6	L2C (1500X) (L2b/UCH-1 & D/UW3/CX)	100%	0.921802841 (L2b/UCH-1); 0.031709167 (D/UW3/CX)	NA

Supplementary Table S3. Pseudo-code description of the primer design algorithm

1.	All the SNP positions were identified on the reference D/UW-3/CX strain in the targeted 100kb region from the 12 strain whole genome MAUVE alignment.
2.	Calculated the frequency of SNPs present in every 100 bp window in the targeted 100kb region.
3.	Identified the 100 bp bins (or windows) that had > 2 SNP positions.
4.	Generated primer3 input files by making sure to exclude the primers being designed on the 100bp bins that had > 2 SNPs in step 3 and maintaining the size of the amplicon between 1100-1300bp. A total of 500 primer3 input files were generated with an increment of 200 bp positions starting from the 100,000 bp position to the 200,000 bp position on the reference D/UW-3/CX genome sequence.
5.	Once all the 500 primer3 input files were generated, a shell script with a do loop that starts at 100000 bp position and increments at every 200 bp until it reached the 200,000 bp position were executed within the primer3 command line executable program in order to generate 500 primer pairs to produce 1.1 to 1.3 kb overlapping amplicons.
